# Transient peripheral compression induces persistent central pain: the acute constriction injury model

**DOI:** 10.3389/fnsys.2026.1962461

**Published:** 2026-10-26

**Authors:** Kaixuan Luo, Xiangjun Feng, Yang Liu, Guanchen Li, Yi Liu, Ping Yang, Yunuo Wang, Yu Sun, Moxian Chen, Shaochun Chen, Lijuan Ao

**Affiliations:** 1 Department of Rehabilitation Therapy, School of Rehabilitation and Yunnan Key Laboratory of Plateau Thermal Medical Rehabilitation and Wellness, Kunming Medical University, Kunming, China; 2 Department of Rehabilitation, The First Affiliated Hospital of Kunming Medical University, Kunming, China; 3 Shenzhen Institute of Advanced Technology (SIAT), Chinese Academy of Sciences, Shenzhen, China; 4 Shanghai Yangzhi Rehabilitation Hospital (Shanghai Sunshine Rehabilitation Center), Tongji University School of Medicine, Shanghai, China

**Keywords:** allodynia, brain imaging, central sensitization, hyperalgesia, neuropathic pain

## Abstract

Traditional neuropathic pain models such as chronic constriction injury (CCI) induce permanent peripheral nerve damage and cannot mimic persistent pain after resolved transient nerve compression. We established an acute constriction injury (ACI) model on male C57BL/6 J mice with 2-h sciatic nerve constriction followed by complete ligature removal, and performed a 250-day multimodal longitudinal comparison of Control, CCI and ACI mice using behavioral pain assays, somatosensory evoked potentials (SEPs), sciatic function index (SFI), nerve histology, muscle atrophy measurement, open field test, and serial 9.4 T pain-evoked BOLD-fMRI. ACI and CCI mice developed comparable long-lasting ipsilateral and delayed contralateral mechanical allodynia and thermal hyperalgesia over the 250-day observation period. Nonetheless, ACI mice achieved robust peripheral nerve structural and functional recovery without muscle atrophy, whereas CCI mice suffered irreversible peripheral nerve lesions. Serial fMRI revealed distinct brain activation trajectories: ACI mice exhibited spatially restricted response reductions relative to Controls and widespread suppression relative to CCI mice at day 3, isolated hypothalamic activation at day 12, and widespread bilateral activation of brainstem descending modulatory networks at day 250; in contrast, CCI mice showed cerebellar and somatosensory cortical activation at early/mid time points and significant deactivation of striatal-limbic circuits at late chronic stages. Anxiety-like behaviors were detected in both CCI and ACI mice at 12 day post-surgery but only in ACI mice at 7 months post-injury. These data support the pain centralization hypothesis: Brief peripheral nerve insult triggers self-maintained central sensitization that sustains chronic pain after peripheral anatomical and functional recovery. The ACI model may facilitate translational research on central-targeted therapeutics for compression-induced persistent neuropathic pain.

## 1 Introduction

Neuropathic pain, arising from lesions or diseases of the somatosensory nervous system, represents one of the most challenging clinical pain conditions. Characterized by spontaneous pain, hyperalgesia, and allodynia (Attal et al., 2023; Baron et al., 2010; van Hecke et al., 2014), neuropathic pain significantly impairs quality of life and imposes substantial socioeconomic burdens (Cohen et al., 2021). However, current therapeutic strategies remain inadequate—meta-analyses indicate that the majority of patients achieve insufficient pain relief with existing pharmacotherapies and neuromodulation techniques, including gabapentin, duloxetine, antidepressants, opioids, transcranial direct current stimulation (tDCS) and repetitive transcranial magnetic stimulation (rTMS) (Soliman et al., 2025). The fundamental challenge lies in our incomplete understanding of neuropathic pain. The causes and mechanisms of neuropathic pain are diverse. Animal models should be designed to mimic the pathophysiological changes in patients with different subtypes of neuropathic pain as closely as possible. Several commonly used laboratory animal models have been proposed via tight ligatures around spinal nerves, transection or loose ligation of either the whole or a part of the sciatic nerve and its branches to create different degrees of denervation (Casaril et al., 2024). The chronic constriction injury (CCI) model, pioneered by Bennett and Xie in 1988, has served as one of the most widely employed models in neuropathic pain research for over three decades (Bennett and Xie, 1988). By applying loose ligatures around the sciatic nerve, CCI reproducibly induces mechanical allodynia, thermal hyperalgesia, and cold allodynia that persist for weeks to months (Casaril et al., 2024). However, a critical limitation of the existing models including CCI is its’ inherent characteristic of permanent peripheral nerve damage—the ligatures which remain in place indefinitely and transections render irreversibly impaired nerve function. This fails to simulate clinical scenarios in a significant proportion of peripheral nerve injury patients—such as those caused by compression (e.g., carpal tunnel syndrome), surgical manipulation, or trauma—are ultimately resolved or relieved. A pivotal question remains unanswered: Does reversible peripheral nerve injury lead to chronic pain, or does pain naturally resolve once the nerve recovers? This question holds profound clinical relevance. In practice, clinicians frequently encounter patients who report persistent pain even after documented neurological recovery—a phenomenon colloquially described as “the nerve healed, yet the pain remained.” Despite its clinical importance, the mechanistic basis for this dissociation remains poorly understood. Critically, animal model that replicate the scenario of “reversible peripheral injury with sustained pain” is still lacking, limiting our ability to investigate the underlying mechanisms.

To address this gap, we developed the acute constriction injury (ACI) model—a novel paradigm in which the sciatic nerve is constricted for 2 h via 3 loose ligatures, after which all ligatures were completely removed. This design simulates a transient nerve compression followed by complete decompression. By systematically comparing ACI, CCI, and Controls, we aimed to: (i) determine whether ACI replicates CCI-like pain behaviors in the early and long-term phase post-injury; (ii) compare the peripheral neuropathic responses following ACI and CCI models postoperatively; (iii) characterize the peripheral neuropathology and central response patterns of the two models via HE staining and BOLD-fMRI, and (iv) compare the temporal profile of affective comorbidity at the early and late time point during the observation period.

## 2 Methods

### 2.1 Experimental design

All experiments were performed on adult male C57BL/6 J mice. After a 7-day acclimatization period following arrival, the animals were randomly allocated into Control, CCI, and ACI groups by means of Microsoft Excel [using the = RAND () function]. Mice underwent model establishment on day 0, pain threshold tests and body weight measurements were conducted on days −1, 3, 6, 9, 12, 15, 18, 30, 60, 90, 120, 150, 180, 210, 240, and 250. Somatosensory evoked potentials (SEPs) of the right hindlimb were recorded on days 1, 9, 12, and 237. Pain-evoked BOLD-fMRI scans were performed on days 3, 12, and 250. Hematoxylin and eosin (HE) staining of the ipsilateral peripheral nerve was carried out on days 1, 3, 12, and 237. The sciatic function index (SFI) was assessed on days −1, 1, 9, 12, and 231. Open field tests (OFT) were conducted on days 12 and 215. In addition, on day 237, the circumference at the knee joint of the right hindlimb was measured for each group to evaluate muscle atrophy. All procedures were approved by the Animal Experimental Ethics Committee of Kunming Medical University (kmmu20230334) and followed the ethical guidelines of the International Association for the Study of Pain (IASP; Zimmermann, 1983). All behavioral tests were performed by investigators blinded to group assignment.

### 2.2 Animals

A total of two hundred and forty adult male C57BL/6 J mice (8–10 weeks old, 22–25 g) were used in the present study. Thirty mice (*n* = 10 per group, 3 groups) were subjected to a panel of pain-related behavioral assays including mechanical allodynia, thermal hyperalgesia, SFI, OFT and body weight monitoring. The remaining four independent experimental cohorts were used exclusively for their respective assays and were not enrolled in any other experimental procedure: Seventy-two mice (*n* = 6 per group per time point, 3 groups, 4 time points) underwent SEP testing, with each mouse tested only once (except the paired ligature +/− recordings, see 2.6). Sixty mice (*n* = 5 per group per time point, 3 groups, 4 time points) were used for HE staining. On day 237, 15 mice from the three experimental groups (*n* = 5 per group) were used for knee circumference measurement. Sixty-three mice (*n* = 7 per group per time point, 3 groups, 3 time points) were used for BOLD-fMRI examinations, with each mouse scanned only once. Immediately after the assigned assay, all mice were deeply anesthetized and humanely euthanized via rapid cervical dislocation.

All animals were obtained from Yunnan University. Mice belonging to the same experimental group were housed together in the same cage (4–5 mice per cage) under a 12-h light/dark cycle, controlled temperature (22–24 °C), and free access to food and water. Animals of different experimental groups were not co-housed within the same cage, which precluded differential access to food or water between injured and control mice and eliminated the potential influence of inter-group competition on behavioral and anatomical outcomes. All experiments were performed during the light phase.

### 2.3 Surgery

The CCI model was established as previously described (Chi et al., 2020; Bennett and Xie, 1988). Briefly, mice were anesthetized with isoflurane (3–5% for induction, 1–2% for maintenance). The right sciatic nerve was exposed at the mid-thigh level. Three ligatures (4–0 silk) were placed around the nerve with approximately 1 mm spacing, each just tight enough to induce visible constriction but not to completely occlude the epineurial blood flow. The incision was closed in layers. Sham-operated mice (Control group) underwent identical surgical procedures without nerve ligation. The ACI model was developed as a modification of CCI. The same surgical approach was performed except untying and removing all ligatures 2 h later (Figure 1). At the beginning, three ligatures were placed identically to CCI and the sciatic nerve as well as the cut tissues were carefully repositioned. After a continuous anesthesia lasting for 2 h, the sciatic nerve was re-exposed and all ligatures were carefully removed. The incision was closed in layers ultimately. To maintain consistency with the ACI group, the Control and CCI group mice were given an additional 2 h of isoflurane maintenance anesthesia after wound closure. Except for the anesthetic, no drugs were administered throughout the entire surgical procedure and subsequent follow-up process.

**Figure 1:**
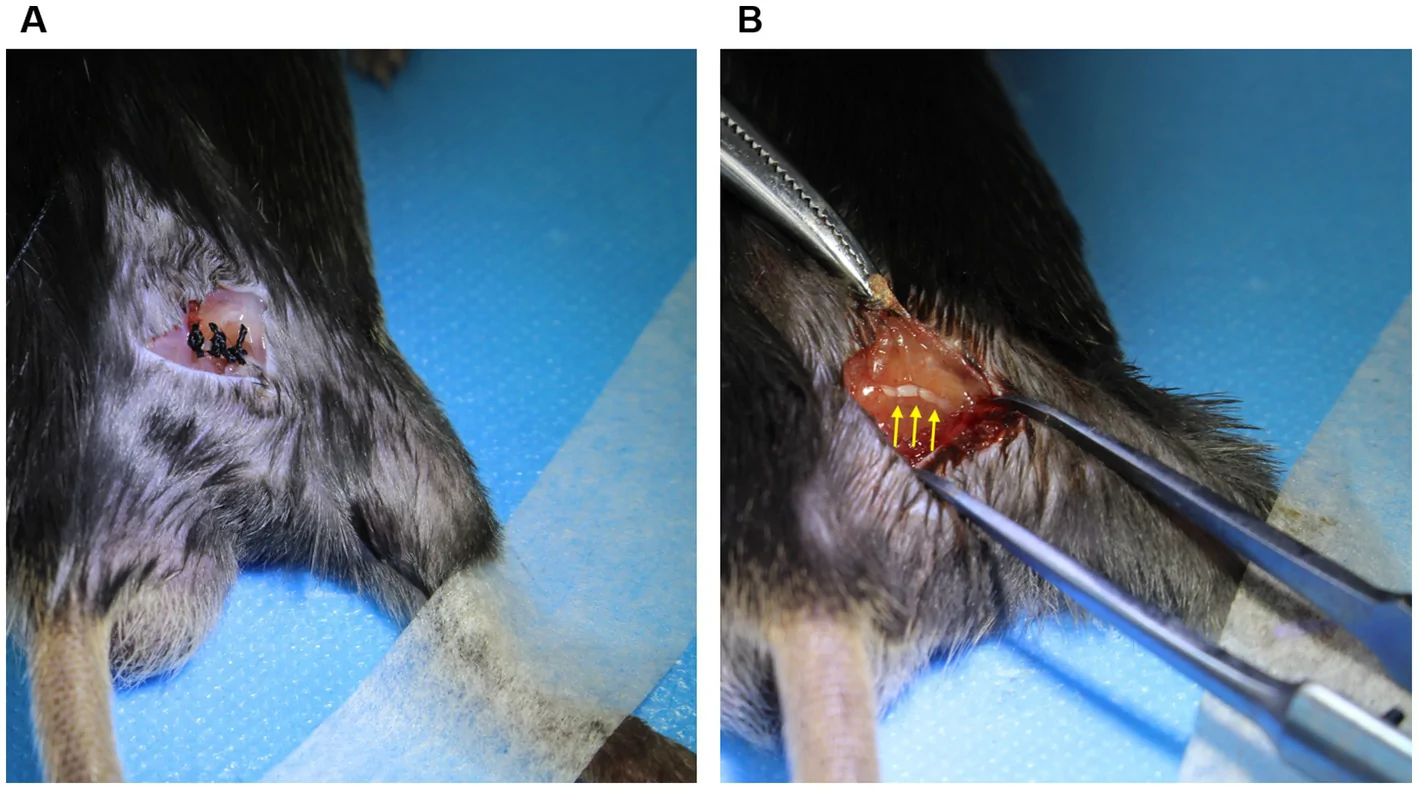
**(A,B)** show the appearance of the sciatic nerve in the ACI group before and after ligature removal, respectively. The yellow arrows indicate the transparent appearance of the ligation site after removal.

The CCI model was originally established in rats by tying four loose ligatures around the sciatic nerve (Bennett and Xie, 1988). In mouse adaptations of this model, the number of loose ligatures is routinely reduced to three or fewer (Chi et al., 2020; Gopalsamy et al., 2019). In the present study, we adopted a standardized configuration of three loose ligatures applied circumferentially to the sciatic nerve for both conventional CCI and the ACI cohorts, corresponding to the standard surgical configuration of the established CCI mouse model, which maximizes comparability with the extensive literature based on this model. The ligatures were removed 2 h after application in ACI mice to model a clinically relevant scenario of transient nerve compression followed by complete decompression. The observed phenotype—pain behavior persisted despite the peripheral anatomical and functional recovery—indicates that this duration may be sufficient to trigger sustained neuropathic pain while preserving nerve reversibility.

### 2.4 Mechanical allodynia

Paw withdrawal threshold (PWT) was measured using the von Frey filament test as previously described (Feng et al., 2021). Mice were placed in individual transparent chambers on a raised mesh floor and allowed to acclimate for 30 min. Von Frey filaments (0.008–2.0 g) were applied to the mid-plantar surface of the ipsilateral and contralateral hindpaw in ascending order. The filament that elicited a paw withdrawal response in ≥2 of 3 trials was recorded as the PWT. There is an interval of 5 ± 10 min between trials.

### 2.5 Thermal hyperalgesia

Paw withdrawal latency (PWL) was assessed using the Hargreaves test (Hargreaves et al., 1988) via a Hargreaves apparatus (No. 37570, Ugo Basile, Comerio, Italy). Mice were placed in individual chambers on a glass surface maintained at 30 °C. A radiant heat source was directed at the plantar surface of the ipsilateral and contralateral hindpaw. The latency to paw withdrawal was recorded (cutoff: 30 s). For each paw, the heat stimulation was applied three times, with a 5 ± 10-min gap between successive applications, and the average value was then calculated. The intensity of the thermal stimulus remained consistent throughout the experiment.

### 2.6 SEPs

SEP recordings were performed on the right hindlimb of mice as previously described (Liao et al., 2021). The animals were anesthetized with an intraperitoneal injection of 1% sodium pentobarbital (40 mg/kg) and fixed in a stereotaxic apparatus. Body temperature was maintained at 37.0 ± 0.5 °C using a heating pad. An incision was made at the middle-distal third of the femur of the right hindlimb to expose the sciatic nerve segment at the surgical site (for CCI mice, the ligature served as a landmark). A pair of stimulating electrodes (2 mm apart) was placed 3 mm distal to the ligation region of the sciatic nerve (in Control and ACI mice, at the corresponding site), with the nerve trunk gently lifted onto the electrode contacts without stretching to deliver constant-current square-wave electrical stimulation (0.2 ms duration, 2 Hz). The stimulus intensity (1 mA) is in line with the previous study for cortical SEP recordings in mice, corresponding to the minimal current producing a visible hindlimb movement (Huang et al., 2018). The active recording electrode was a homemade L-shaped stainless steel wire, which was connected to the amplifier via a stainless steel clip. This electrode was inserted into a circular burr hole (diameter 0.8–1.0 mm) drilled over the hindlimb representation area of the left primary somatosensory cortex (coordinates: AP -0.94 mm, ML − 1.6 mm relative to bregma) and was gently advanced to make light contact with the dura mater. The reference electrode (stainless steel needle) was placed subcutaneously at the nasal bridge, and the ground electrode was inserted subcutaneously in the dorsum. Upon stimulation, at least 100 evoked responses were continuously recorded with a bandpass filter of 10–2000 Hz. For each time point, two runs of 100 averaged responses were performed with a 5-min interval, and the two averages were further averaged. The latency was defined as the time (ms) from the onset of the stimulus artifact to the first peak (positive or negative). The amplitude (μV) was measured from the positive peak to the negative peak. To clarify the effect of the ligature on SEPs, in CCI mice, after SEP recording at days 1 and 9 postoperatively, the ligature was carefully removed, and the second batch SEP recordings were performed 5 min later.

### 2.7 SFI

Functional recovery of the sciatic nerve was assessed via SFI (Inserra et al., 1998) with some modification. Footprints were collected by dipping the hindpaws in ink and allowing mice to walk across a sheet of paper through an acrylic track (80 cm) which is opaque on both sides and transparent on top. The following parameters were measured: Print length (PL): Distance from the heel to the third toe, toe spread (TS): Distance from the first to the fifth toe. These measurements were obtained from the experimental (E) and normal (N) paws. SFI was calculated as: SFI = −51.25 × (EPL - NPL)/NPL + 1118.91 × (ETS - NTS)/NTS – 7.57. All tests were conducted at the same time of the day (09:00 a.m.) by the same researcher.

To intuitively observe the effects of surgery on the hindlimbs of mice, the condition of the bilateral hindlimbs was observed from bottom to top at days −1, 1, 9, 12, and 231 postoperatively. Briefly, mice from the three groups were placed in separate compartments on a transparent glass platform, and the right hindlimb and toes were observed to assess changes over time.

### 2.8 Muscle atrophy

The right hindlimbs of the mice were immersed in tissue fixative solution. After adequate fixation, the skin and hair were removed, and the fixative was thoroughly blotted dry with absorbent paper. The circumference of the knee joint was measured using a tape measure (Figure 2B). Each sample was measured three times, and the average value was calculated.

**Figure 2:**
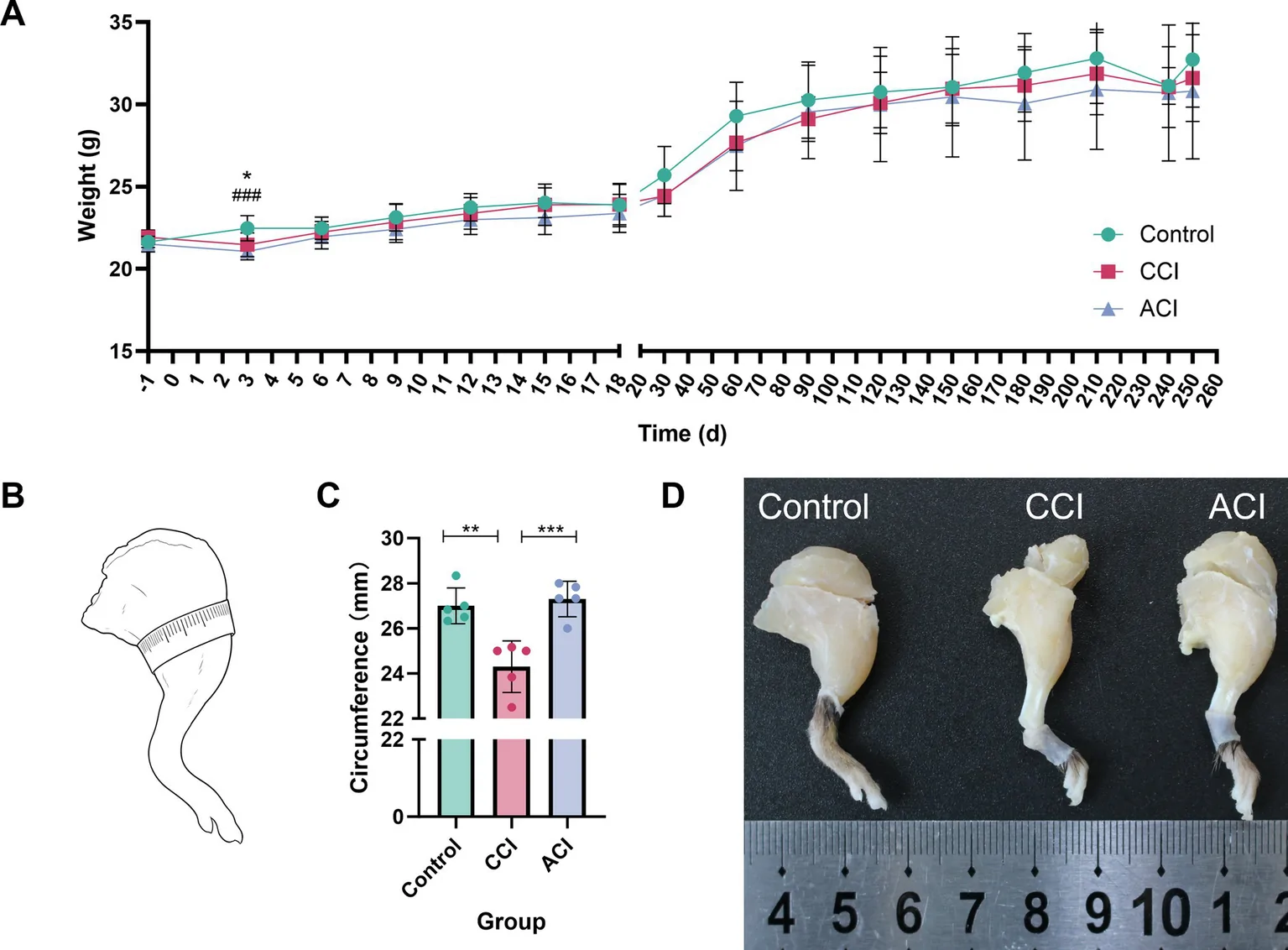
**(A)** Changes in body weight over time in each group of mice (*n* = 10). *p* values for comparisons between the CCI group and the Control group are indicated as: ^*^p < 0.05, ^**^p < 0.01, ^***^p < 0.001. Comparisons between the ACI group and the Control group are indicated as: *^#^p* < 0.05, *^##^p* < 0.01, *^###^p* < 0.001. Comparisons between the ACI group and the CCI group are indicated as: *^^^p* < 0.05, *^^^^p* < 0.01, *^^^^^p* < 0.001. **(B–D)** Measurement of the knee joint circumference of the right hindlimb in mice at 237 days post-surgery. **(B)** Schematic diagram of the measurement method, **(C)** quantitative results of the circumference (*n* = 5 per group), **(D)** representative images comparing samples from different groups.

### 2.9 Histological examination

Mice were perfused with paraformaldehyde (4%) under anesthesia. The sciatic nerve at the lesion site was harvested, fixed, and embedded in paraffin. Sections (5 μm) were cut and stained with HE.

### 2.10 OFT

The open field test was performed as previously described (Luo et al., 2025) to evaluate spontaneous locomotor activity and anxiety-like behavior in mice. The apparatus consisted of a square arena made of opaque plastic, measuring 40 cm (length) × 40 cm (width) × 40 cm (height). The floor was divided into two virtual zones: a central zone (occupying the central 25% of the total area) and a peripheral zone (the remaining area adjacent to the walls). Prior to testing, all mice were acclimated to the testing room for at least 60 min to minimize environmental stress. At the start of each trial, a single mouse was gently placed into the center of the arena and allowed to explore freely for a total duration of 5 min. All behaviors were recorded by an overhead camera and analyzed using ANY-maze video-tracking software (Stoelting Co., Wood Dale, IL, USA). Between consecutive trials, the arena was thoroughly cleaned with 70% ethanol and dried with paper towels to eliminate any olfactory cues from the previous animal, thereby preventing potential bias in subsequent tests.

### 2.11 MRI acquisition and data analysis

#### 2.11.1 MRI acquisition and stimulus paradigm

All MRI data were acquired using a 9.4 T preclinical MRI system (uMR9.4 T, United Imaging Healthcare, Shanghai, China). During EPI-BOLD scanning, body temperature of mouse was maintained at 37 ± 1 °C and respiratory rate at 100–120 breaths per minute by adjusting the water circulating system and anesthesia workflow. Mice were initially anesthetized in an induction chamber with 3–4% isoflurane delivered via 1 L/min medical air. Upon loss of paw withdrawal reflex, the animals were rapidly transferred and secured in a custom non-metallic MRI-compatible stereotaxic holder, whose head-fixation geometry is fully analogous to that of a standard rodent stereotaxic apparatus, to rigorously restrict cranial motion during the entire MRI session. Isoflurane anesthesia was maintained through a nose mask integrated into the holder assembly, and the concentration was titrated down and stabilized at 0.75 ± 0.25% before the initiation of functional MRI sequences. Once the mice that under stable anesthesia state was properly positioned in the scanner, scout images were acquired to assess positioning quality. For coregistration purposes, an anatomical reference scan was performed using an FSE 2D T2-weighted sequence with the following parameters: TE/TR = 46.48/3600 ms, flip angle = 180°, FOV = 15 × 19 mm^2^, 38 slices of 0.4 mm thickness, matrix size = 208 × 208, bandwidth = 220 Hz/pixel, echo train length = 13, voxel size = 0.09 × 0.09 × 0.40 mm^3^, number of averages = 4, scan time = 3 min 11 s. Functional MRI data were acquired with a single-shot echo planar imaging (EPI) sequence using the following parameters: TE/TR = 14/2000 ms, flip angle = 60°, FOV = 15 × 19 mm^2^, 38 slices of 0.4 mm thickness, matrix size = 96 × 96, bandwidth = 3,470 Hz/pixel, echo train length = 68, voxel size = 0.20 × 0.20 × 0.40 mm^3^, scan time = 8 min 52 s. Slices were positioned identically to those in the anatomical scan.

The task paradigm used a block design with mechanical stimulation. After an initial baseline resting period (R0) of 20 TRs (40 s), the sequence comprised 6 alternating cycles of rest blocks (R1–R6) and mechanical stimulation blocks (S1–S6). Each rest and stimulation block lasted 20 TRs (40 s). For mechanical pain stimulation, the hook side of a hook-and-loop fastener was firmly fixed onto a flat wooden board, which was positioned 0.5 cm directly behind the plantar surface of the mouse’s right hindpaw (the operated side). An inelastic nylon thread was loosely looped around the mouse’s ankle, and the free end of the thread was passed through a pre-calibrated circular hole on the board. To ensure consistent stimulation parameters across all animals, all pulling operations were performed by the same experimenter, who was blinded to group allocation. A metronome was used to maintain the stimulation frequency at 1 Hz, and pre-marked graduations on the thread ensured that the displacement of each pull remained essentially constant at 1 cm. Each pull was terminated immediately upon reaching the pre-determined position and perceiving a slight resistance threshold. This threshold had been validated in three naive control mice before the formal experiments to ensure that stable contact between the plantar surface and the hook surface was reliably established in every animal, thereby inducing reproducible mechanical nociceptive stimulation.

#### 2.11.2 Image processing of MRI data

The imaging data were processed using the Joint Neuroanalysis Module within the United Imaging MR postprocessing workstation (uWS MR). For analysis, the first time point (R0) was removed, and all subsequent processing and statistical analyses were performed on the remaining time series. The preprocessing pipeline included data sorting, format conversion, motion correction, eddy current correction, skull stripping, coregistration of structural and functional images, normalization to standard space, and spatial smoothing. In terms of fMRI head motion quality control, 6-parameter rigid-body correction was performed on all functional time series. For all datasets, the cumulative translational motion across the entire scanning session did not exceed 0.075 mm, and the cumulative rotational motion did not exceed 0.3°. All 6 head motion parameters were incorporated as nuisance covariates into the general linear model to regress out residual signal artifacts associated with head motion. Subsequently, within the uWS MR Joint Neuroanalysis Module, a general linear model (GLM) was constructed according to the task paradigm to perform first-level task-based fMRI analysis for each mouse individually. This allowed estimation of stimulus-related BOLD signal changes and generation of individual-level maps of significant activation or deactivation. Concurrently, quality control (QC) plots of head motion were produced for each subject to evaluate data quality. The primary comparison focused on alterations in brain regional excitability between the CCI and ACI modeling groups relative to the Control group (*n* = 7 per group). Group-level analysis was also conducted using the uWS MR Joint Neuroanalysis Module, employing whole-brain voxel-wise statistics. Based on predefined contrasts, two-sample t-tests were applied to the individual-level results to assess between-group differences in task-related BOLD responses. Multiple comparison correction was performed using Gaussian random field (GRF) correction, with a voxel-level threshold of *p* < 0.005 and a cluster-level threshold of *p* < 0.05. The final outputs included uncorrected second-level statistical maps, corrected significance maps, significant cluster images, and tables of identified significant brain regions. Anatomical labeling of significant regions was performed according to a standard mouse brain atlas, and for each significant cluster, the number of voxels, peak t-value, and peak Allen coordinates were extracted. Anatomical regions were defined based on the complete whole-brain delineation atlas integrated via the HiDiver multimodal registration pipeline (Johnson et al., 2023).

### 2.12 Statistics

All continuous measurement data are presented as mean ± standard deviation (SD). Normality of data distribution was assessed using the Shapiro–Wilk test, and homogeneity of variances was verified via Levene’s test prior to inter-group statistical comparisons. No data points were excluded from the final statistical analysis, and all collected experimental datasets were fully included for statistical computation. For independent, single-timepoint datasets with equal variances, one-way analysis of variance (one-way ANOVA) was performed to detect significant overall differences across the three experimental groups. For datasets that violated the homogeneity of variance assumption, the non-parametric Kruskal–Wallis one-way analysis of variance by ranks was adopted for multi-group overall comparison instead. Once a statistically significant global difference was confirmed, post-hoc pairwise inter-group comparisons were conducted: the least significant difference (LSD) test was applied following significant one-way ANOVA, while Dunn’s test with adjusted *p* values was implemented after a significant Kruskal–Wallis H test. All one-way ANOVA and Kruskal-Wallis H analyses were performed using IBM SPSS Statistics 25.0 software. All longitudinal repeated measurements, including mechanical allodynia, thermal hyperalgesia, SFI and body weight, were analyzed using a two-way mixed-design repeated-measures analysis of variance (RM-ANOVA) with a full factorial structure. The within-subject factor consisted of all consecutive observational time points covering pre-surgery baseline and the post-surgery follow-up period, while the between-subject factor included three experimental groups: Control, CCI and ACI. When the sphericity assumption was violated as assessed by Mauchly’s test, the Greenhouse–Geisser correction was applied to adjust the degrees of freedom. After a statistically significant group-by-time interaction effect was confirmed, post-hoc simple effect analyses restricted to each individual time point were performed for pairwise intergroup comparisons, and all *p* values from multiple comparisons were corrected using Tukey’s honestly significant difference method to control the family-wise Type I error rate. Two-way RM-ANOVA analyses were performed through GraphPad Prism version 10.1.2 software. A two-tailed *p* value < 0.05 was defined as the threshold for statistical significance for all statistical tests. All experimental graphs were generated with GraphPad Prism version 10.1.2 software. Sample size was calculated in advance using G*Power 3.1.9.2. For the one-way ANOVA across three groups, the effect size *f* = 0.70 was determined based on the mean and standard deviation of PWL outcomes obtained from the preliminary experiment. The α was set to 0.05 and power = 0.80, the calculated minimum required total sample size was 24. The corresponding sample size (n) is detailed in respective figure legends.

## 3 Results

### 3.1 Mechanical allodynia

#### 3.1.1 Ipsilateral hindlimb

The first evaluation of right hindlimb PWTs (*n* = 10 per group, Figure 3A) was carried out 1 day before surgery (day −1). There was no significant intergroup difference in the Control, CCI, and ACI groups in terms of the ipsilateral baseline PWTs values, indicating consistent baseline mechanical sensitivity across all experimental groups. On postoperative day 3, the PWTs of the right hindlimb were significantly reduced in the CCI group 0.0148 ± 0.0105 g and ACI group 0.0092 ± 0.0038 g compared to the Control group 0.4200 ± 0.0632 g, with statistical significance at *p* < 0.0001. Over the subsequent observation period, the PWTs of the operated hindlimb remained persistently low in both the CCI and ACI groups, which differed significantly from the Control group at all time points examined. No significant differences in PWTs were observed between the CCI and ACI groups throughout the entire experimental course.

**Figure 3:**
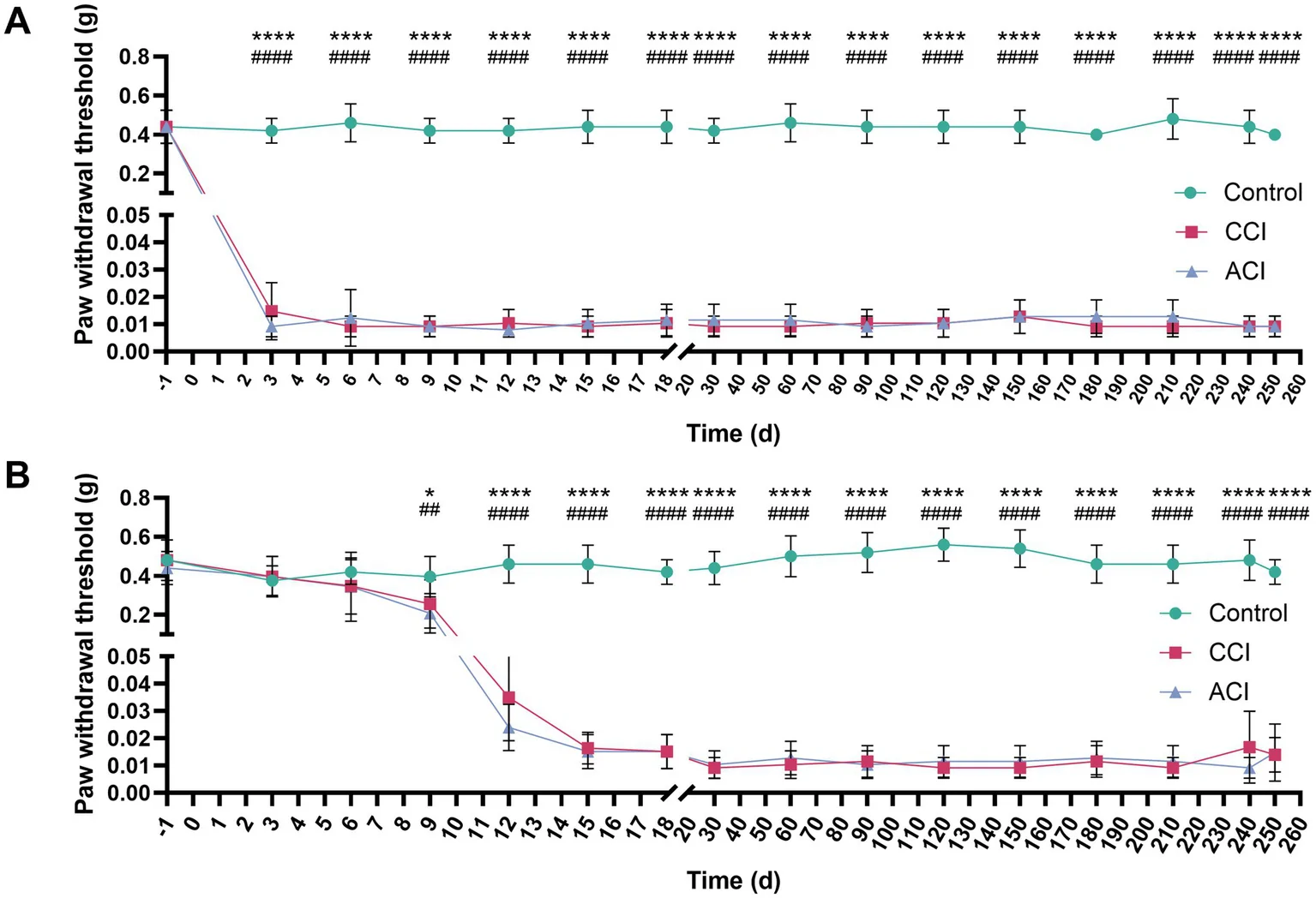
Changes in paw withdrawal threshold of the ipsilateral (right, **A**) and contralateral (left, **B**) hindpaw over time in the Control, ACI, and CCI groups (*n* = 10 per group). *p* values for comparisons between the CCI group and the Control group are indicated as: ^*^p < 0.05, ^**^p < 0.01, ^***^p < 0.001, ^****^p < 0.0001. Comparisons between the ACI group and the Control group are indicated as: *^#^p* < 0.05, *^##^p* < 0.01, *^###^p* < 0.001, *^####^p* < 0.0001. Comparisons between the ACI group and the CCI group are indicated as: *^^^p* < 0.05, *^^^^p* < 0.01, *^^^^^p* < 0.001, *^^^^^^p* < 0.0001.

#### 3.1.2 Contralateral hindlimb

Preoperative baseline PWTs (*n* = 10 per group, Figure 3B) of the contralateral (left) hindpaw were assessed on day −1 in all groups. The baseline values were 0.4800 ± 0.1033 g, 0.4800 ± 0.1033 g, and 0.4400 ± 0.0843 g for the Control, CCI, and ACI groups, respectively, with no significant intergroup differences, indicating consistent contralateral baseline mechanical sensitivity across groups. From postoperative days 3 to 6, no significant changes in PWTs were observed in the CCI or ACI groups compared to the Control group, although a tendency toward decreased thresholds was noted. On postoperative day 9, the contralateral PWTs in the CCI and ACI groups were 0.2560 ± 0.1239 g and 0.2080 ± 0.1012 g, respectively, both significantly lower than that of the Control group 0.3960 ± 0.1041 g. Over the subsequent 20 days, the contralateral PWTs in both injury groups further declined to low levels. By day 30, the thresholds reached their nadir, with values of 0.0092 ± 0.0038 g in CCI group and 0.0104 ± 0.0051 g in ACI group, and remained at low levels until the end of the behavioral testing period (day 250). From day 9 onward, the contralateral PWTs in both the CCI and ACI groups were lower than those in the Control group, whereas no significant difference was found between the CCI and ACI groups at any time point.

### 3.2 Thermal hyperalgesia

#### 3.2.1 Ipsilateral hindlimb

No significant difference was detected in terms of PWLs (*n* = 10 per group, Figure 4A) of the ipsilateral hindpaw among three groups at day −1, indicating a preoperative consistent baseline. On postoperative day 3, the PWLs in both the CCI group 5.0700 ± 1.0958 s and ACI group 4.6083 ± 1.2915 s were lower than that in the Control group 9.5733 ± 1.3926 s (*p* < 0.001). From day 3 to day 12, the PWLs in the CCI and ACI groups continued to decline and thereafter remained at low levels throughout the observation period. At all time points except for −1 day, the ipsilateral PWLs in both injury groups were shorter than those of the Control group (*p* < 0.0001), whereas no significant difference was observed between the CCI and ACI groups at any time point.

**Figure 4:**
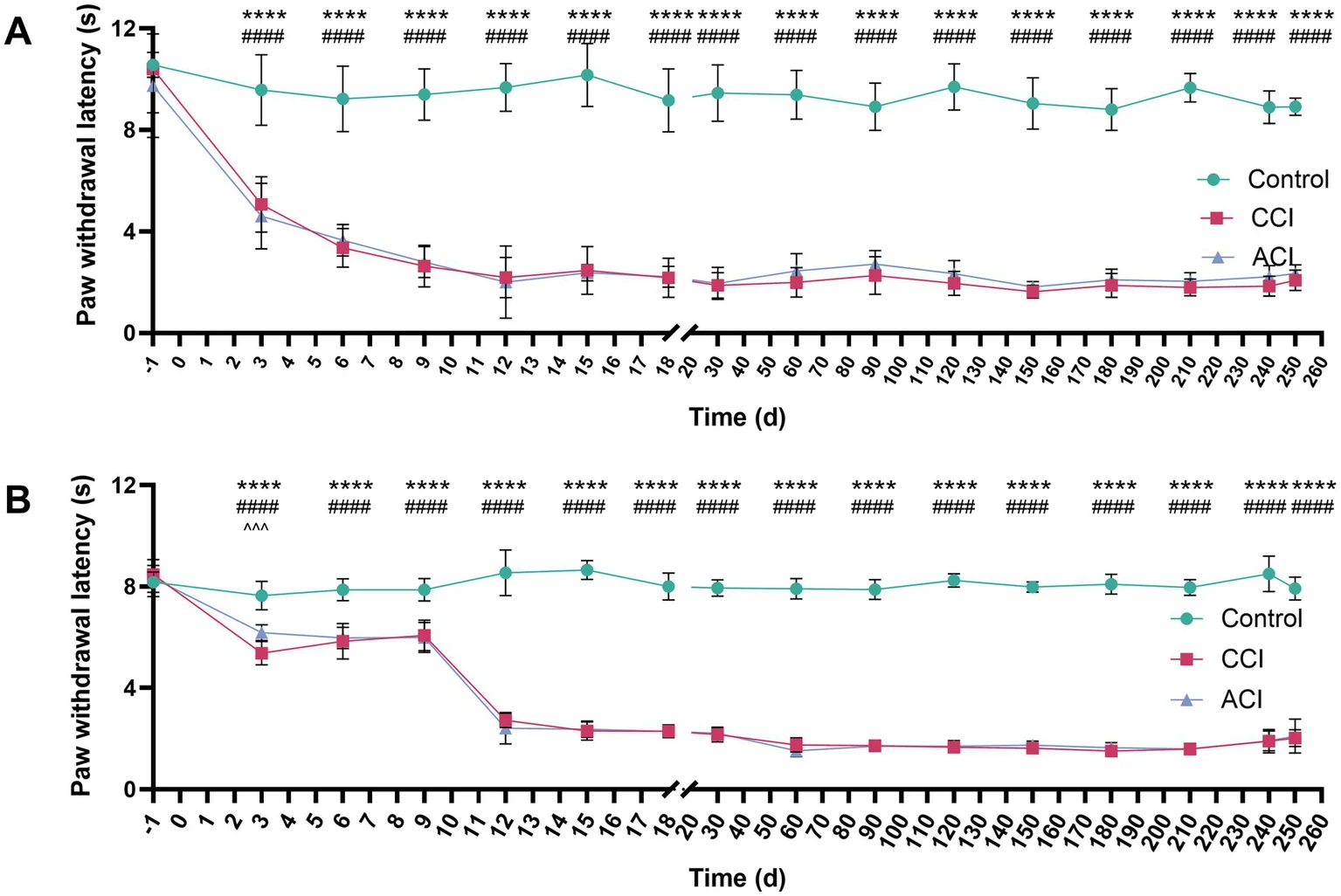
Changes in Paw withdrawal latency of the ipsilateral (right, **A**) and contralateral (left, **B**) hindpaw over time in the Control, ACI, and CCI groups (*n* = 10 per group). *p* values for comparisons between the CCI group and the Control group are indicated as: ^*^p < 0.05, ^**^p < 0.01, ^***^p < 0.001, ^****^p < 0.0001. Comparisons between the ACI group and the Control group are indicated as: *^#^p* < 0.05, *^##^p* < 0.01, *^###^p* < 0.001, *^####^p* < 0.0001. Comparisons between the ACI group and the CCI group are indicated as: *^^^p* < 0.05, *^^^^p* < 0.01, *^^^^^p* < 0.001, *^^^^^^p* < 0.0001.

#### 3.2.2 Contralateral hindlimb

Preoperative baseline PWLs (*n* = 10 per group, Figure 4B) of the contralateral (left) hindpaws showed no significant differences among groups. On postoperative day 3, both the CCI and ACI groups developed significant thermal hyperalgesia, as indicated by reduced PWLs. At this time point, the PWL in the ACI group 6.1833 ± 0.3136 s was significantly higher than that in the CCI group 5.3783 ± 0.4678 s, although both were significantly lower than the Control group 7.6500 ± 0.5655 s. From day 3 to 9, the contralateral PWLs in both injury groups stayed at a plateau, and then further decreased by day 12, remaining at low levels thereafter until the end of the observation period. Throughout the experiment, the PWLs in both the CCI and ACI groups were shorter than those in the Control group at all time points except for −1 day. No difference was observed between the CCI and ACI groups at any other time point except for day 3.

### 3.3 SEPs

To assess the effect of the ligature itself on SEPs, SEP recordings of CCI mice were performed before ligature removal-CCI (ligature +) group and after ligature removal-CCI (ligature -) group within the first 9 postoperative days as described in the Methods. On day 1, SEP latencies in the CCI (ligature +) group were significantly prolonged (Figure 5A) compared to the Control and ACI groups, whereas no significant differences were observed between the Control and ACI groups, or between the CCI (ligature +) and CCI (ligature -) group. Regarding SEP amplitudes, the CCI (ligature +), CCI (ligature -), and ACI groups all exhibited significantly reduced amplitudes relative to the Control group. Notably, ligature removal in CCI mice resulted in a significant increase in amplitude as demonstrated in Figure 5B. On day 9, the latency pattern (Figure 5C) remained consistent with that observed on day 1: CCI (ligature +) mice showed prolonged latencies compared with the Control and ACI groups (*p* < 0.05), with no significant differences detected between the Control and ACI groups or between the CCI (ligature +) and CCI (ligature -) groups. Latency of CCI (ligature -) prolonged significantly compared to the Control group. With respect to amplitudes on day 9 (Figure 5D), CCI (ligature +) group displayed significantly lower values than those in the ACI and Control groups, whereas no significant changes were detected between the Control and ACI groups or between the two CCI subgroups. From day 12 onward, ligature removal was technically infeasible due to marked adhesions between the sciatic nerve and surrounding tissues, as well as progressive nerve degeneration. Therefore, no further ligation removals were performed. At days 12 and 237, CCI (ligature +) mice exhibited prolonged SEP latencies (*p* < 0.001 Figures 5E and 5G) and reduced amplitudes (*p* < 0.05 Figures 5F and 5H\) compared to both the Control and ACI groups, while no differences in either parameter were found between the Control and ACI groups (*p* > 0.05). Representative SEP waves of mice in each group at each time point are shown in Figure 5I.

**Figure 5:**
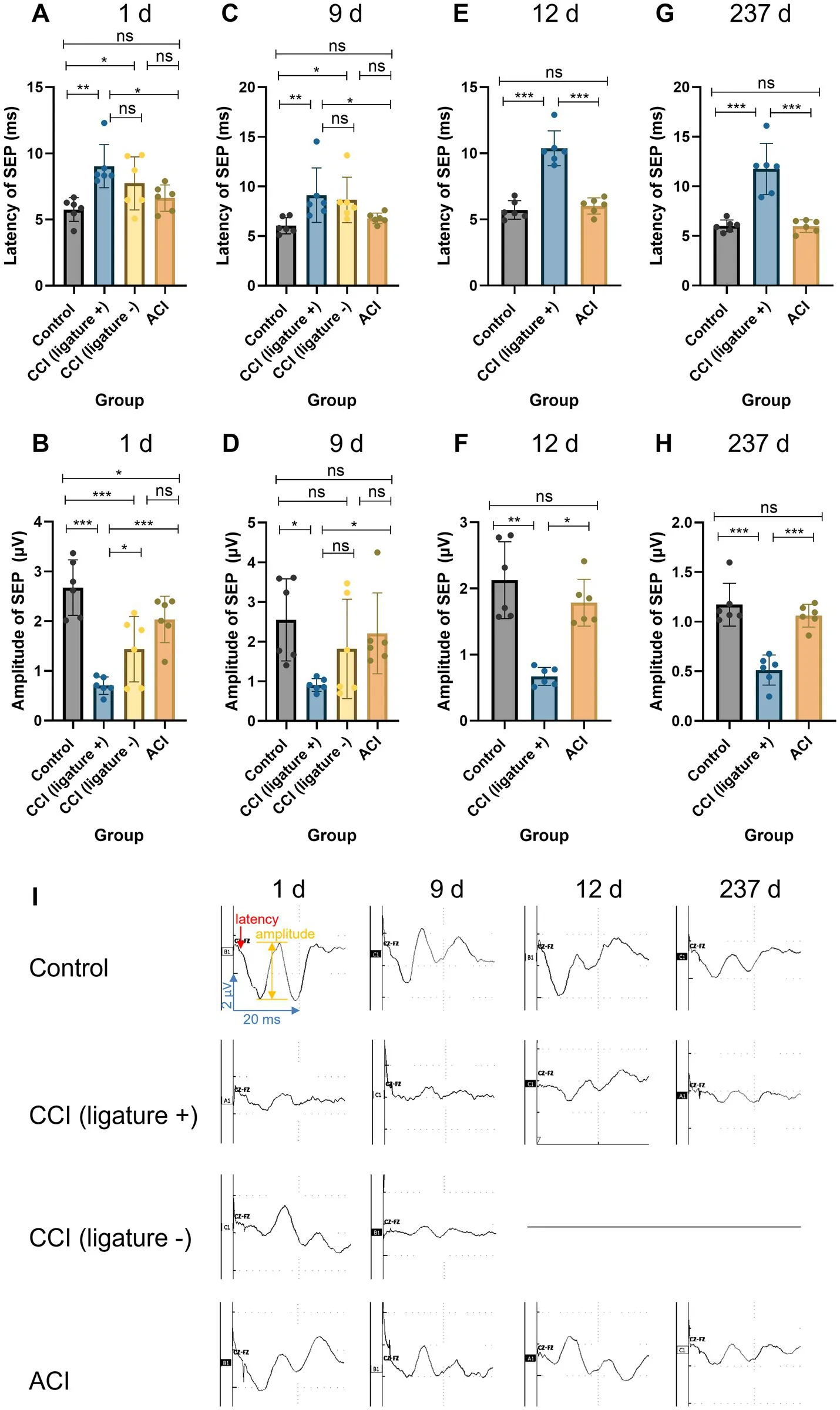
Latency and amplitude comparisons for each group of mice at 1 d (panels **A,B**), 9 d (panels **C,D**), 12 d (panels **E,F**), and 237 d (panels **G,H**) after surgery (*n* = 6 per group). Panel **I** displays representative SEP waveforms at each time point for all groups. The waveforms shown under CCI (ligature +) and CCI (ligature −) conditions correspond to the same mouse. In the first trace of the Control group at 1 d post-operation, the horizontal scale bar defines a time interval of 20 ms, and the vertical scale bar defines an amplitude of 2 μV. As an example, the red arrow marks the manually measured latency, and the yellow double-arrow line marks the amplitude. All other traces in this figure share the identical 20 ms time base and 2 μV amplitude scaling for consistent comparison across groups and time points. ^*^p < 0.05, ^**^p < 0.01, ^***^p < 0.001, ns, no significant difference.

### 3.4 SFI

For the baseline consistency, the preoperative (day −1) SFI values (*n* = 10 per group) of the right hindlimb in the Control, CCI, and ACI groups were −7.2313 ± 1.1618, −7.5700 ± 0.0000, and −7.5700 ± 0.0000, respectively, with no significant differences among the three groups as shown in Figure 6A. At postoperative day 1, SFI values in the CCI and ACI groups decreased to −90.2657 ± 4.8821 and −80.0329 ± 13.1243, respectively, which were lower (*p* < 0.0001) than that of the Control group −7.1674 ± 0.8723. On day 9, the SFI in the ACI group recovered to −44.4084 ± 20.6562, which was significantly lower than that of Control group −7.8342 ± 0.8356 whereas higher than CCI group −91.8955 ± 3.817. By day 12, the ACI mice achieved an SFI value of −9.3119 ± 2.8888, which was not significantly different from that of the Control group. The CCI group, however, maintained a low SFI at −91.3447 ± 3.4073. At day 231, CCI mice demonstrated a SFI of −91.7478 ± 2.3436, significantly lower than both ACI -10.0613 ± 3.7904 and Control mice −7.5700 ± 0.0000. From postoperative day 12 onwards, the SFI values in the ACI group were not significantly different from those in the Control group. To visually evaluate the functional recovery of the right sciatic nerve, toe posture was monitored in each group (Figure 6B–F; Supplementary Videos 1–5). Preoperatively, all mice displayed natural spreading of the right hindpaw toes and normal weight-bearing (Figure 6B; Supplementary Video 1). At postoperative day 1, both CCI and ACI mice exhibited toe flexion, adduction, eversion, and avoidance of weight bearing on the affected limbs (Figure 6C; Supplementary Video 2). On day 9 (Figure 6D; Supplementary Video 3), ACI mice showed varying degrees of toe spreading and ground contact. By day 12 (Figure 6E; Supplementary Video 4) and 231 (Figure 6F; Supplementary Video 5), the toe posture of the affected hindpaw in the ACI group exhibited a normal pattern, whereas the CCI group consistently displayed an abnormal pattern of toe flexion, adduction, and eversion throughout the entire observation period.

**Figure 6:**
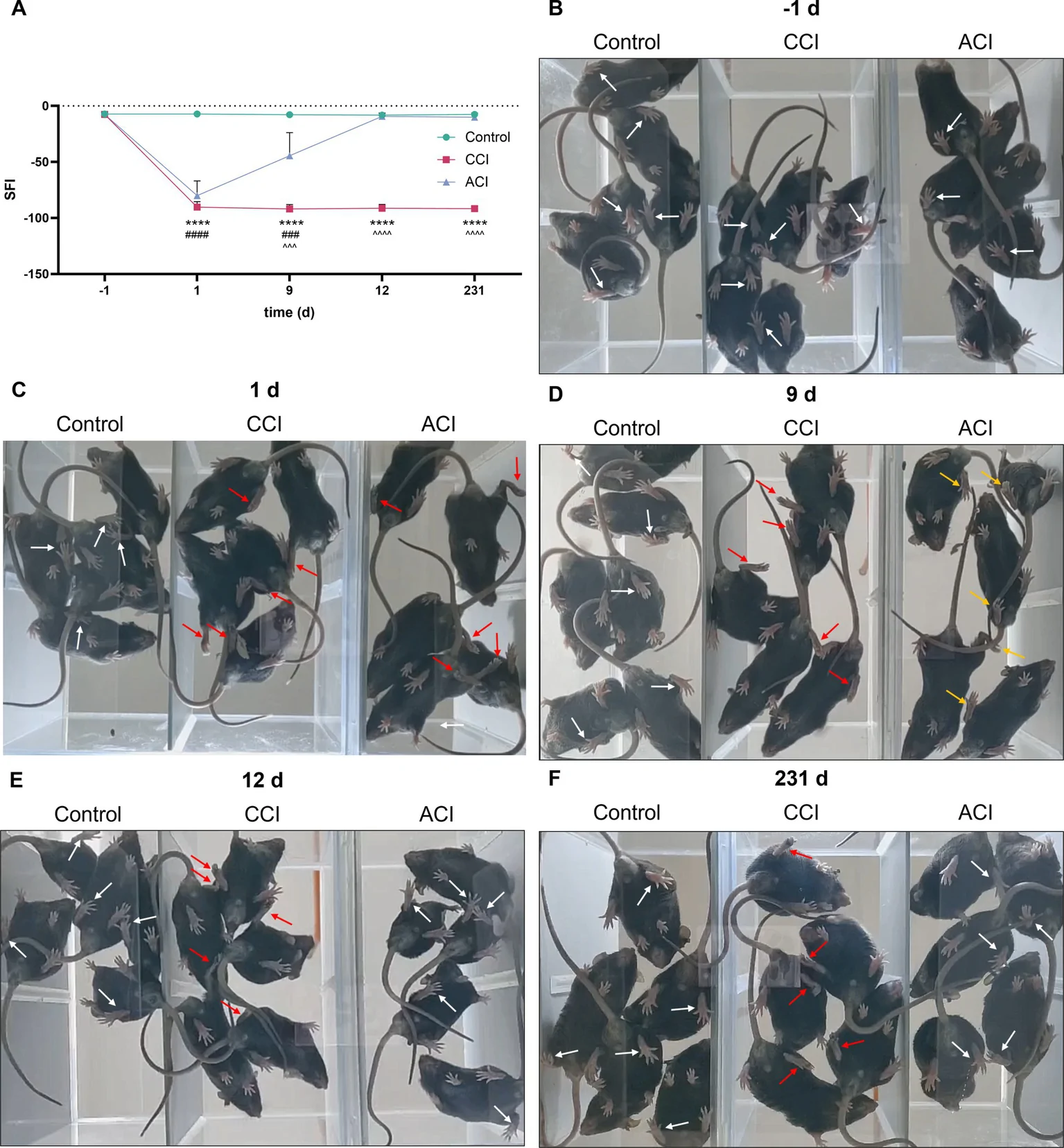
**(A)** SFI over time in the Control, ACI, and CCI groups (*n* = 10 per group). *p* values for comparisons between the CCI group and the Control group are indicated as: ^*^p < 0.05, ^**^p < 0.01, ^***^p < 0.001, ^****^p < 0.0001. Comparisons between the ACI group and the Control group are indicated as: *^#^p* < 0.05, *^##^p* < 0.01, *^###^p* < 0.001, *^####^p* < 0.0001. Comparisons between the ACI group and the CCI group are indicated as: *^^^p* < 0.05, *^^^^p* < 0.01, *^^^^^p* < 0.001, *^^^^^^p* < 0.0001. **(B–F)** Appearance of the ipsilateral (right) hindlimb of mice in each group at day −1 (preoperative, panel **B**) and at days 1 **(C)**, 9 **(D)**, 12 **(E)**, and 231 **(F)** after surgery. Normal paw appearance is indicated by white arrows (as in panel **B**). In CCI mice, the right hindpaw exhibited obvious plantar flexion, eversion, and toe adduction at day 1 after surgery, and these abnormalities persisted throughout the observation period (red arrows). In ACI mice, the paw appearance was similar to that of CCI mice at day 1 (red arrows in panel **C**), but showed marked improvement by day 9 (yellow arrows), with toes opening and weight-bearing to varying degrees. At days 12 and 231, the right paw appearance of ACI mice was almost identical to the normal pattern of Control mice (white arrows).

### 3.5 OFT

As illustrated in Figure 7, on day 12 after surgery, both CCI and ACI mice exhibited shorter duration in the center zone (*p* < 0.01) and longer duration in the peripheral zone (*p* < 0.01) compared to the Control group. The number of entries into the center zone reduced (*p* < 0.01), while number of entries into the peripheral zone increased (*p* < 0.01) in both CCI and ACI groups compared to the Control mice. The total distance moved, total duration of movement and mean speed decreased (*p* < 0.01, *p* < 0.05, *p* < 0.01, respectively), whereas the total duration of immobility increased (*p* < 0.05) in both CCI and ACI groups compared to the Control group. No significant differences were observed between the CCI and ACI groups in any of the above parameters. The above findings demonstrate that mice in both the CCI and ACI groups displayed characteristic anxiety-like behaviors along with decreased locomotor activity.

**Figure 7:**
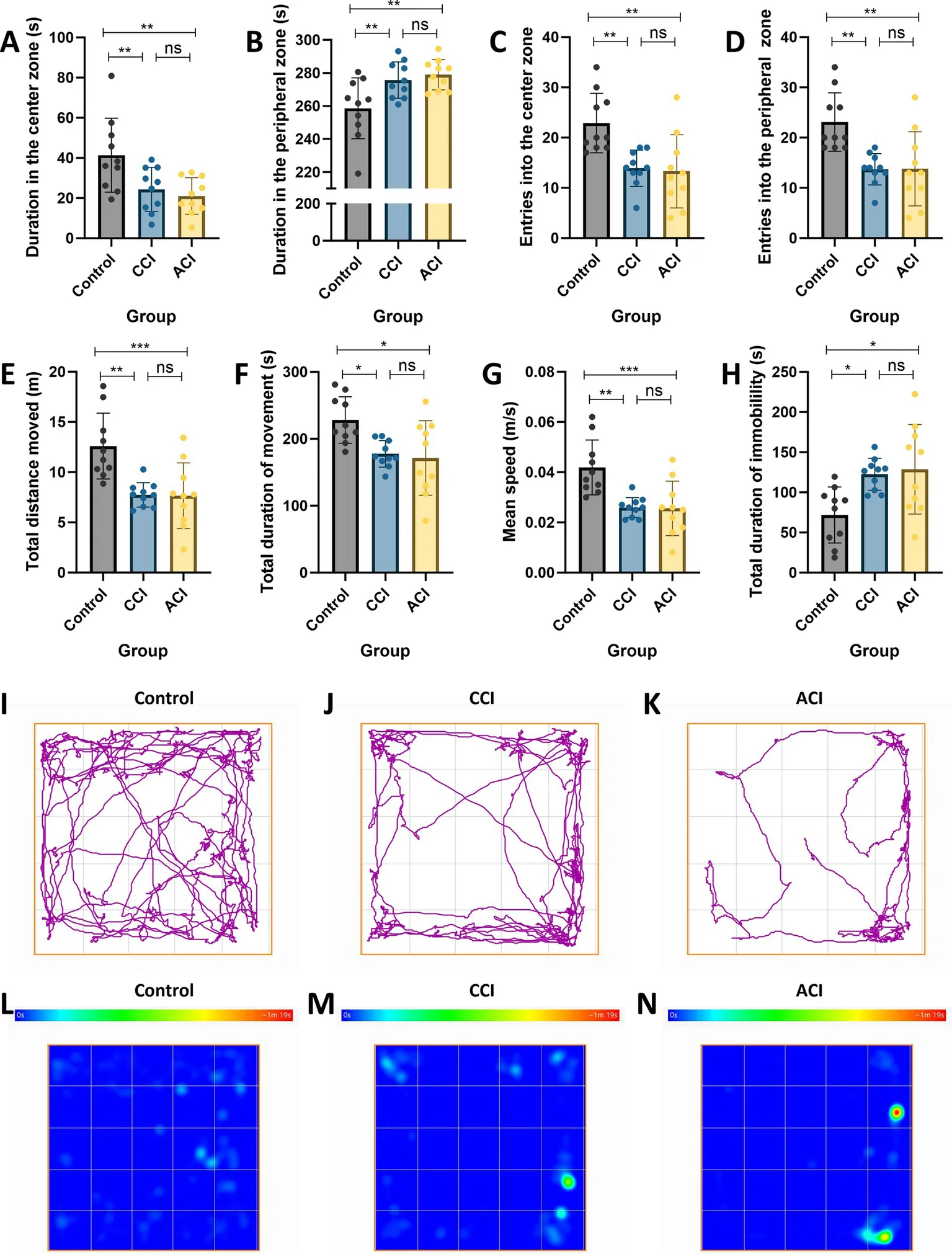
OFT results of Control, CCI, and ACI mice at 12 day after surgery (panels **A–H**, *n* = 10 per group). Panels **I–K** show representative track plots for each group, and panels **L–N** show the corresponding representative heatmaps. The track plots illustrate the movement trajectory of the animal’s center point during the testing session. The heatmaps indicate the percentage of time that the animal’s center point spent in different areas of the open field. ^*^p < 0.05, ^**^p < 0.01, ^***^p < 0.001, ns, no significant difference.

The OFT results on day 215 (Figure 8) revealed that ACI mice spent significantly shorter duration in the center zone and longer duration in the peripheral zone than both CCI and Control mice. In contrast, no differences were found between the CCI and Control groups in these measures (*p* > 0.05). There were no significant differences detected among the three groups in the number of entries into the center as well as the peripheral zone, the total distance moved, total duration of movement, mean speed and the total duration of immobility. Together, these findings indicate that in contrast to CCI mice, ACI mice still exhibited anxiety-like behavior at day 215 after surgery. Notably, this behavioral change occurred in the absence of any intergroup difference in the number of entries into either zone or locomotor measures mentioned above, indicating that the persistent reduction in center-zone dwell time in ACI mice was not secondary to reduced locomotor activity or exploratory drive.

**Figure 8:**
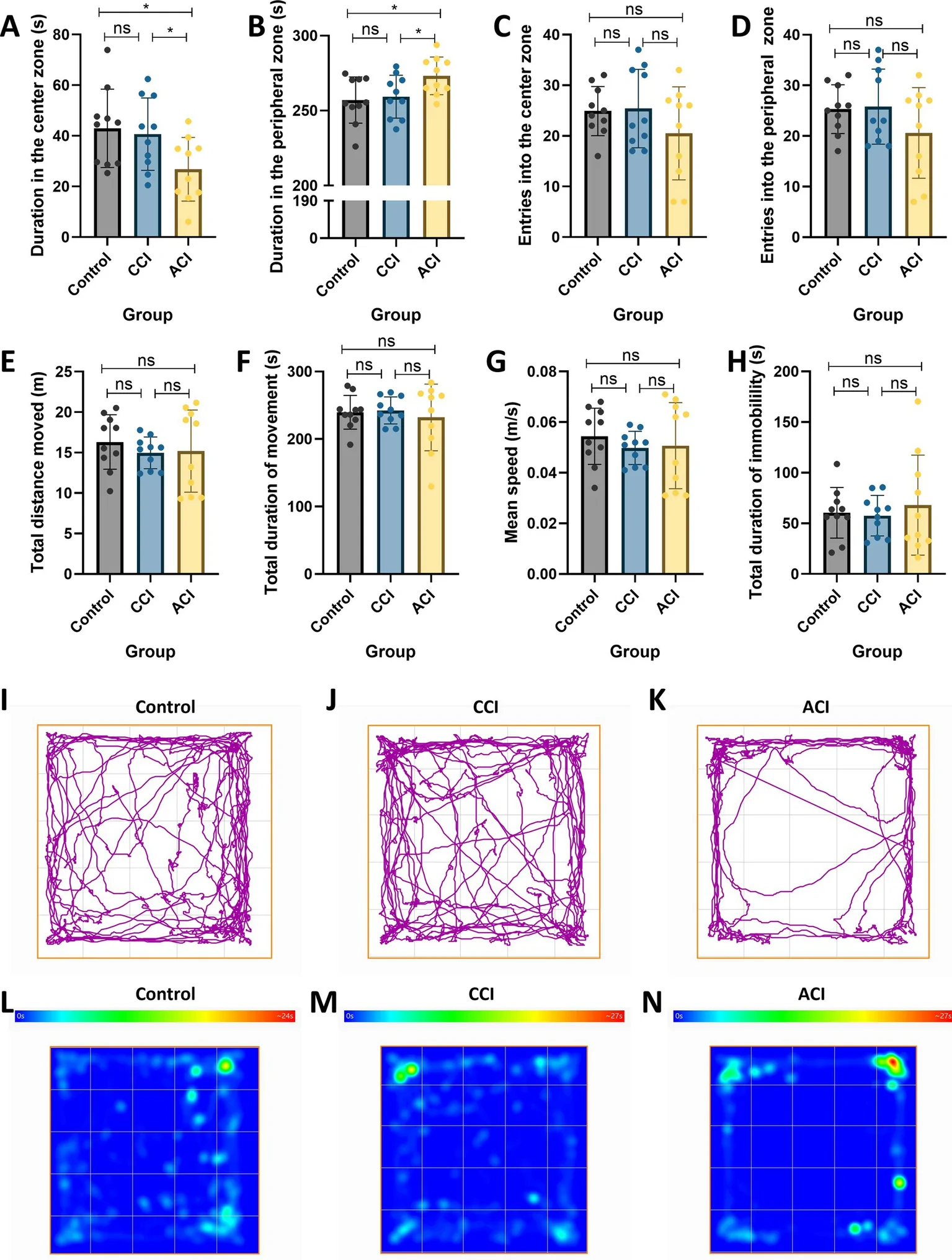
OFT outcomes for Control, CCI, and ACI mice at 215 day post-surgery (panels **A–H**, *n* = 10 per group). Panels **I–K** show representative track plots for each group, and panels **L–N** show the corresponding representative heatmaps. ^*^p < 0.05, ^**^p < 0.01, ^***^p < 0.001, ns, no significant difference.

### 3.6 Histological examination of the sciatic nerve

Histological assessment of the ipsilateral sciatic nerve was performed at 1, 3, 12, and 237 days post-surgery (Figure 9). In the CCI group, nerve fiber disruption and structural disorganization were evident around the ligature site at all time points. Inflammatory cell infiltration (lymphocytes and granulocytes) was pronounced at 1 and 3 days, accompanied by hemorrhage and scattered necrotic debris. By day 12, dense connective tissue encapsulation surrounded the ligatures, with extensive inflammatory infiltration and vacuolization of nerve fibers. At 237 day, the nerve architecture remained disorganized, with persistent connective tissue hyperplasia and chronic inflammation. In contrast, the ACI group exhibited only mild hemorrhage and moderate inflammatory infiltration at day 1, which largely resolved by day 3. At day 12, occasional vacuolization and mild inflammatory cells were observed, and by 237 day, the nerve structure appeared intact with no significant abnormalities. The Control group showed minimal changes across all time points, with only occasional mild inflammatory infiltration or slight hemorrhage in the early postoperative phase, and no overt structural alterations. The representative gross appearance of the affected nerves of mice in each group at 237 day post-surgery is shown in Figure 10.

**Figure 9:**
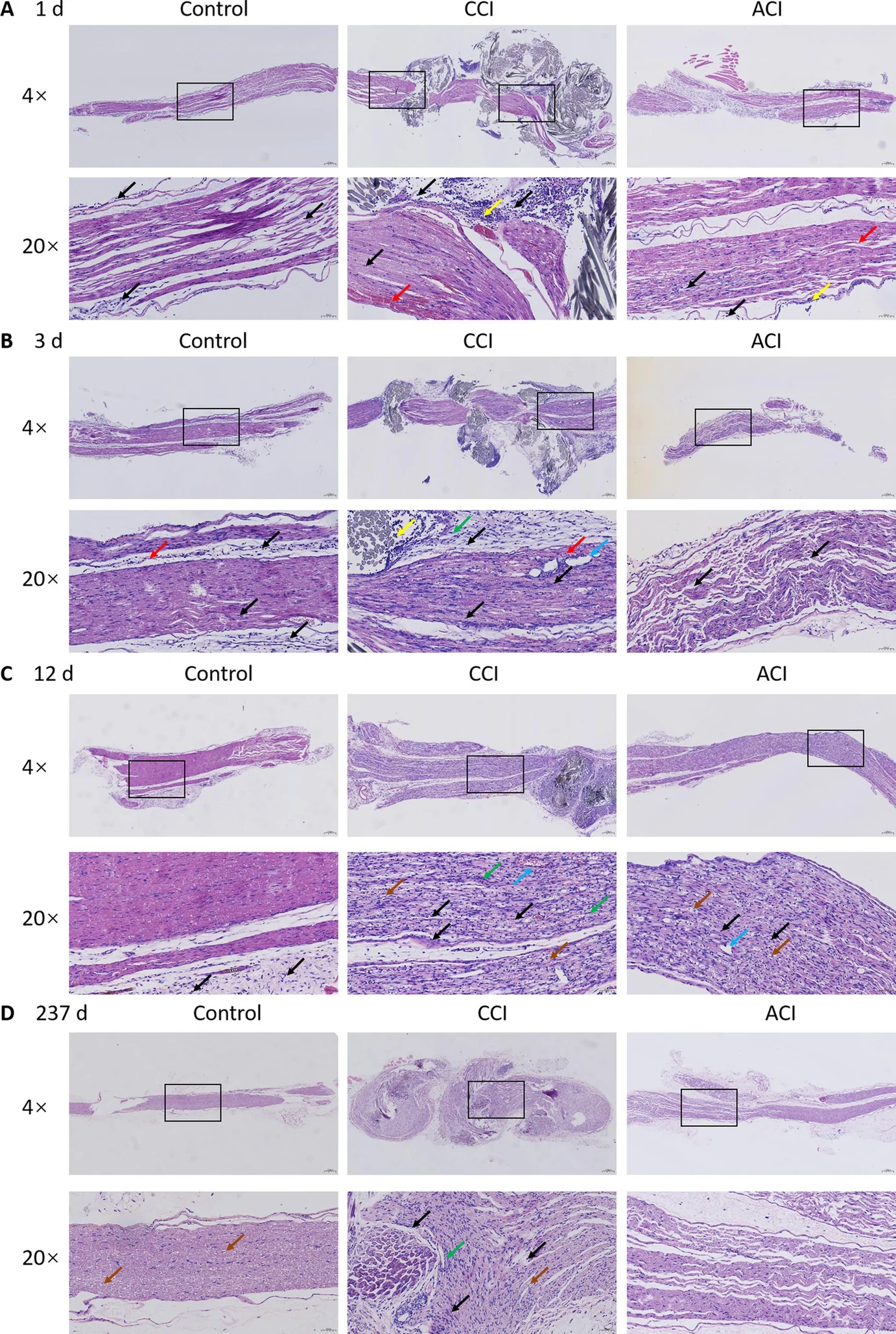
Representative HE-stained sections of mouse sciatic nerves (right side) at 1 (**A**, *n* = 5 per group), 3 (**B**, *n* = 5 per group), 12 (**C**, *n* = 5 per group), and 237 (**D**, *n* = 5 per group) days post-surgery. In the CCI group, the ligature knot induced nerve fiber disruption and disorganization, accompanied by abundant and persistent infiltration of lymphocytes and granulocytes, hemorrhage, and necrotic debris. In later stages, connective tissue proliferation encapsulated the foreign body, and some nerve fibers showed vacuolar degeneration. In the Control group, nerve fibers were generally well-arranged with intact structures, showing only occasional mild inflammatory cell infiltration and vacuolar changes, without obvious foreign body reaction. The ACI group exhibited acute inflammatory responses initially, but the inflammation did not become chronic. Black arrows indicate infiltration of inflammatory cells (lymphocytes, granulocytes, etc.), green arrows indicate connective tissue proliferation, brown arrows indicate vacuolar degeneration of nerve fibers, blue arrows indicate vascular dilation, yellow arrows indicate necrotic cell debris, and red arrows indicate hemorrhage.

**Figure 10:**
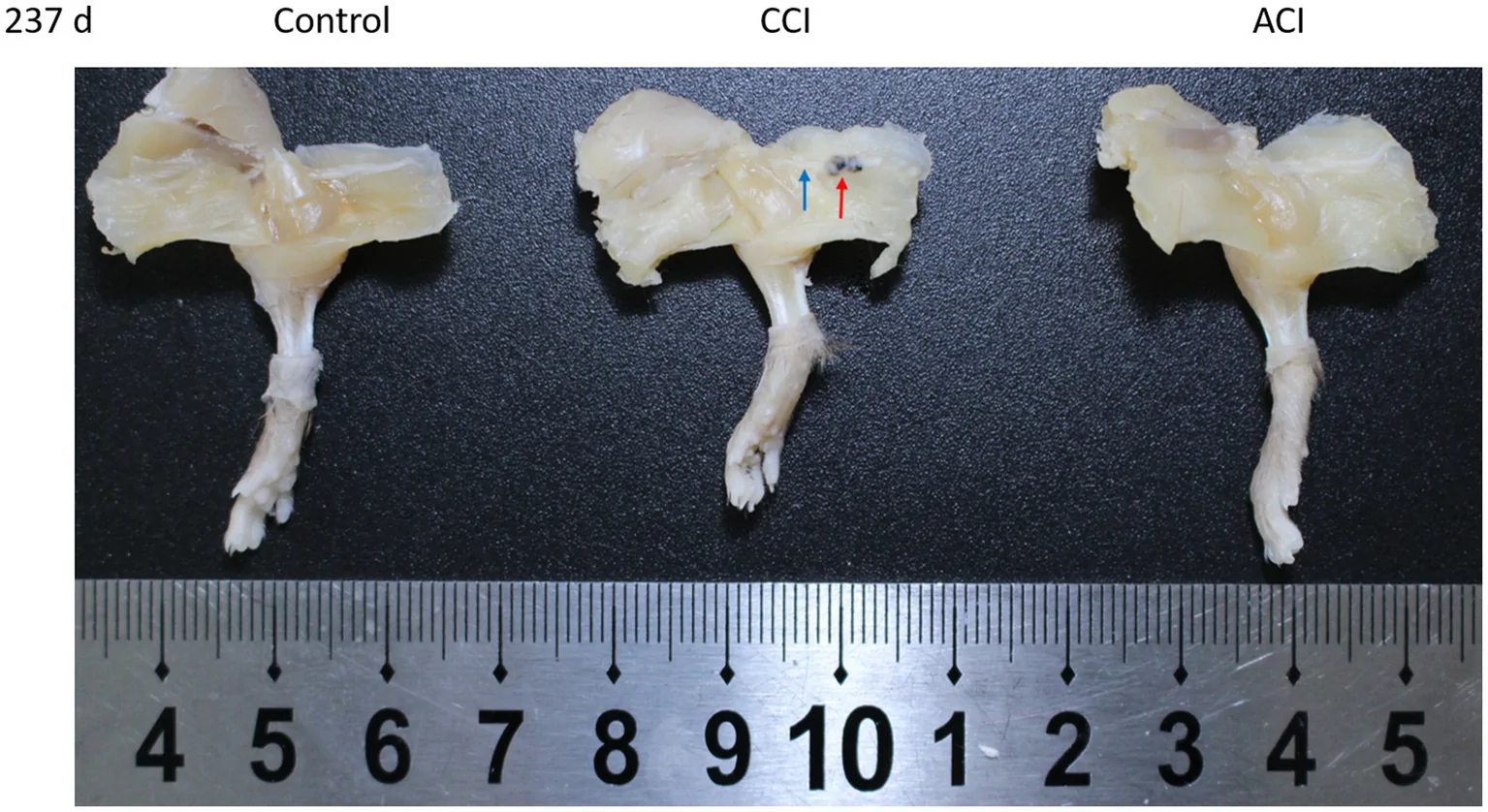
Macroscopic findings of the right sciatic nerves in each group at 237 d (*n* = 5 per group). In the CCI group, the ligature knot was clearly visible on the sciatic nerve (red arrow), and the nerve around the foreign body showed hyalinization (yellow arrow).

### 3.7 Body weight and muscle atrophy

As illustrated in Figure 2A, before operation (day −1), there were no differences in body weight among three groups (*p* > 0.05), indicating baseline comparability. At day 3 after modeling, the body weights of the CCI and ACI groups were 21.4700 ± 0.7196 g and 21.0600 ± 0.5125 g, respectively, were both significantly lower than that of the Control group 22.4700 ± 0.7718 g. The weight differences observed at this time point were most likely caused by the surgery. From then until day 250, no differences in body weight were observed among three groups (*p* > 0.05), indicating the consistency of nutritional levels among groups. Regarding the muscle atrophy (Figures 2B–D) at day 237, circumference at the knee joint of the right hindlimb in the Control, CCI, and ACI groups was 27.0000 ± 0.7906, 24.3000 ± 1.1390, and 27.3000 ± 0.7853 mm, respectively. The CCI group exhibited significantly atrophic circumference at the knee joint than both the Control and ACI groups, whereas no difference was found between the Control and ACI groups (*p* > 0.05), indicating that CCI mice developed significant right-hindlimb atrophy, whereas no such atrophy was observed in ACI mice.

### 3.8 Pain-evoked BOLD fMRI responses

At postoperative day 3, the CCI group exhibited significant activation peak in the left cerebellar vermis compared to the Control group, (Supplementary Table 1; Figure 11A). In contrast, the ACI group showed significant decreased responses in the left temporal association area and left primary visual cortex upon mechanical stimulation of the right hindpaw (Supplementary Table 2; Figure 11B). Compared with the CCI mice, the ACI group exhibited widespread reductions in BOLD signals in response to pain stimulation, involving bilateral cortical regions (sensorimotor, limbic, and multimodal association areas), subcortical structures (basal ganglia, midbrain motor nuclei, parabrachial and vestibular nuclei), and the cerebellum (vermis and interposed nuclei), along with concomitant decreases in major white-matter commissural pathways (corpus callosum, cingulum bundle, and hippocampal commissure) as illustrated in Supplementary Table 3 and Figure 11C.

**Figure 11:**
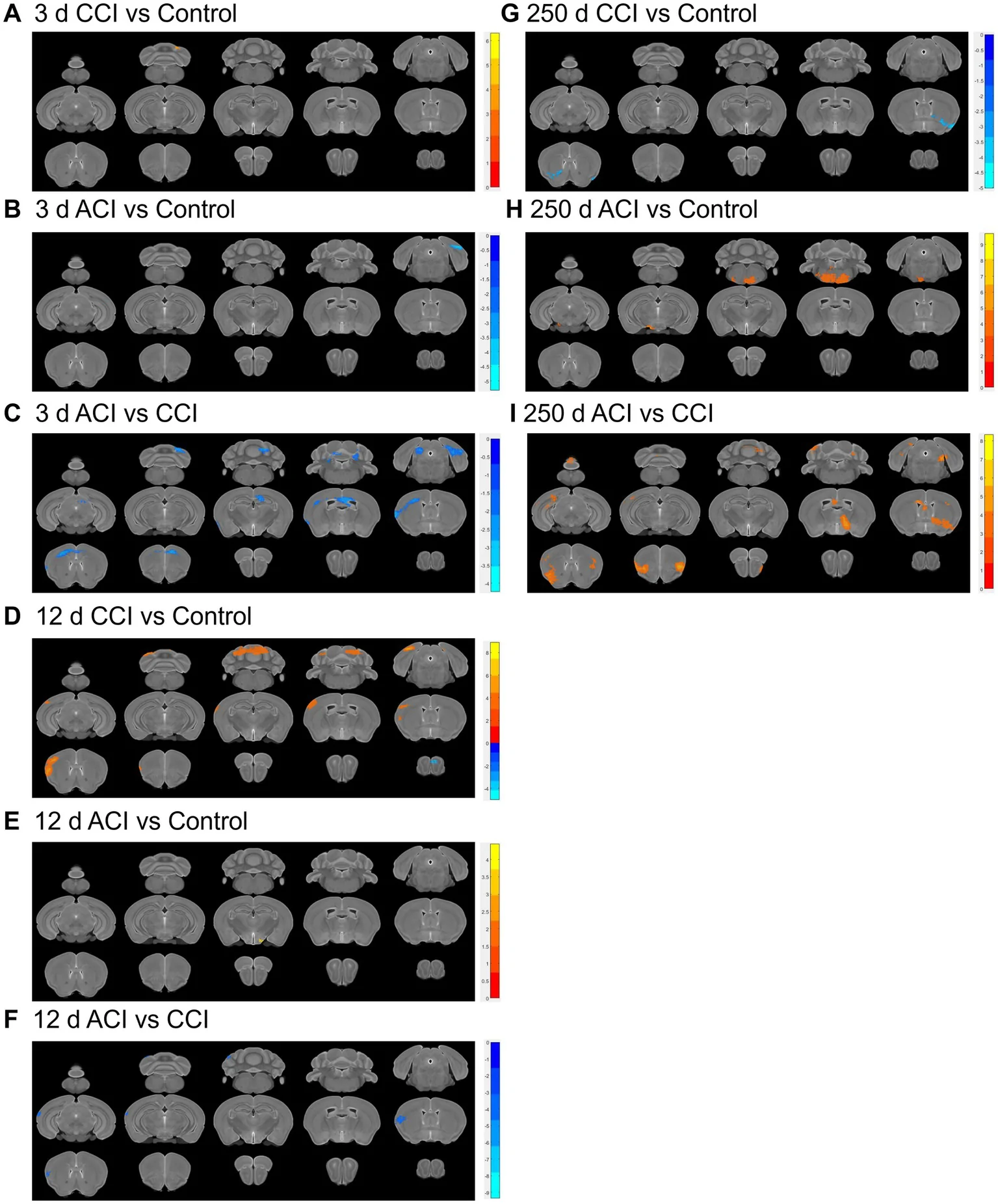
Comparison of BOLD responses to mechanical pain stimulation of the right hindpaw among groups at 3 d (**A–C**, *n* = 7 per group), 12 d (**D–F**, *n* = 7 per group), and 250 d (**G–I**, *n* = 7 per group). The colorbar represents t-values of the statistical image.

At day 12, significant BOLD signal increases were observed bilaterally in the retrosplenial cortex and the cerebellar vermis, as well as in the left central nucleus of the inferior colliculus in the CCI mice. On the right hemisphere, widespread cortical activations were detected, involving the primary visual area, the agranular insular area, gustatory areas, primary motor cortex, primary somatosensory cortex (including the barrel field), secondary somatosensory area, and auditory areas. In contrast, the left olfactory bulb displayed a significant decreased response to mechanical stimulation of the contralateral (right) hindpaw, suggesting a cross-modal suppression effect (Supplementary Table 4; Figure 11D). In the ACI group, significant activation peaks were observed in the hypothalamus, specifically in the ventromedial hypothalamic nucleus (VMH) and the tuberal nucleus (TN) as demonstrated in Supplementary Table 5 and Figure 11E. ACI mice exhibited reduced BOLD signals in the right sensorimotor, insular, temporal-association, and striatal regions under mechanical pain stimulation compared to CCI mice (Supplementary Table 6; Figure 11F).

At 250 d after surgery, the CCI group showed prominent BOLD signal decreases predominantly confined to four functional networks during right hindpaw mechanical pain stimulation (Supplementary Table 7; Figure 11G). First, bilateral olfactory-related regions, including the piriform cortex and endopiriform nucleus, together with the right olfactory tubercle showed reduced BOLD signals. In addition, the striatal complex was widely engaged, as evidenced by bilateral deactivation of the dorsal striatum, along with left-sided involvement of the fundus of the striatum and right-sided involvement of the nucleus accumbens. Third, the left bed nucleus of the stria terminalis, a key node of the extended amygdala showed reduced activation. Finally, reduced BOLD signal was observed in the right anterior commissure. In ACI mice, activation was observed in the bilateral brainstem reticular formation and raphe nuclei, the bilateral facial motor nuclei, and the right-sided trigeminal (principal sensory and spinal), vestibular, and cochlear nuclei, together with the right hypothalamus (including the mammillary body and lateral hypothalamic area) and the right substantia nigra pars reticulata (Supplementary Table 8; Figure 11H). Interestingly, compared with CCI mice, ACI group exhibited significant BOLD activation peaks in response to right hindpaw mechanical pain across multiple bilateral brain regions (Supplementary Table 9; Figure 11I), including the orbital, insular, gustatory, piriform, motor, somatosensory, auditory, visual, temporal-association, and retrosplenial cortices; the hippocampal formation (CA1, dentate gyrus, entorhinal and subicular areas); the basal ganglia (striatum, nucleus accumbens, substantia innominata); the thalamic and hypothalamic nuclei and the zona incerta; midbrain structures (inferior colliculus, pedunculopontine nucleus, cuneiform nucleus, lateral lemniscus); the cerebellum (vermis, flocculonodular region); and major white-matter tracts (corpus callosum, cingulum bundle, fornix, anterior commissure).

## 4 Discussion

### 4.1 Principal findings

The principal findings can be summarized as follows: (i) Despite the brevity of the compression and the complete removal of all ligatures, ACI mice developed robust and persistent mechanical allodynia and thermal hyperalgesia on the ipsilateral hindlimb that were comparable to those observed in CCI mice and endured for the entire 250-day observation period; (ii) Both ACI and CCI mice developed delayed, progressive contralateral mechanical allodynia and thermal hyperalgesia, indicating bilateral central sensitization; (iii) While CCI mice exhibited persistent sciatic nerve damage, ACI mice showed robust peripheral nerve recovery, including normalization of SEPs, restoration of SFI, absence of muscle atrophy, and intact nerve architecture by day 237; (iv) BOLD-fMRI revealed divergent supraspinal activation trajectories between ACI and CCI mice; (v) Anxiety-like behaviors persisted in ACI mice at 7 months post-injury, whereas CCI mice showed emotional recovery despite ongoing nociceptive behaviors. Collectively, these findings reveal a striking paradox: A brief, reversible peripheral nerve injury can engender chronic pain that is maintained long after the peripheral trigger has resolved, thereby offering an important nuance to the prevailing view that persistent neuropathic pain requires sustained peripheral nerve damage.

### 4.2 The ACI model: bridging a critical translational gap

Widely adopted classic neuropathic pain models, including CCI (Bennett and Xie, 1988), spared nerve injury (Decosterd and Woolf, 2000) and spinal nerve ligation (Ho Kim and Mo Chung, 1992) models, rely on permanently damaged peripheral nerves to induce stable pain phenotypes—thereby failing to capture a prevalent clinical scenario in which the inciting compression is transient and subsequently resolved. Clinical conditions such as carpal tunnel syndrome, tarsal tunnel syndrome and radiculopathy frequently involve mechanical nerve injury, where surgical decompression is an important intervention option (Middleton and Anakwe, 2014; Rodríguez-Merchán and Moracia-Ochagavía, 2021; Toci et al., 2023). Importantly, a proportion of patients who experienced surgery develop chronic neuropathic pain (Kuffler et al., 2025). The ACI model was specifically designed to address this translational gap. By constricting the sciatic nerve for only 2 h before complete ligature removal, the ACI paradigm isolates the effects of transient compression from those of persistent structural damage, thus providing a platform to investigate the mechanisms of sustain pain despite the recovery of peripheral injury.

### 4.3 Peripheral recovery versus persistent pain: insights into the centralization hypothesis

A core finding of our study is the observable dissociation between the anatomical as well as functional recovery of peripheral nerve and sustained chronic pain in ACI mice. ACI mice exhibited functional limb recovery as early as day 12, the normalized nerve conduction reflected by SEP recordings by day 9, and intact nerve morphology with marked resolution of inflammatory responses at day 237, with no detectable denervation-related muscle atrophy. In sharp contrast, CCI mice displayed persistent nerve conduction abnormalities and obvious muscular atrophy throughout the experimental period. Notably, despite comprehensive peripheral functional and structural recovery, ACI mice retained severe mechanical allodynia and thermal hyperalgesia equivalent to CCI mice until day 250. This striking dissociation implies that persistent chronic pain in the ACI model is independent of ongoing peripheral nociceptive input, suggesting that central sensitization is a major mechanism maintaining post-injury chronic pain (Latremoliere and Woolf, 2009; Woolf, 2011). Central sensitization manifests as heightened neuronal excitability and strengthened synaptic efficacy across spinal and supraspinal pain circuits, accompanied by a spectrum of adaptive changes: synaptic transmission remodeling, glial activation, and reshaping of the neuroinflammatory microenvironment (Basbaum et al., 2009; Latremoliere and Woolf, 2009). In the context of the ACI model, we propose that transient 2-h nerve compression triggers initial nociceptive input and acute neuroinflammation, which may initiate and sustain central sensitization, leading to long-term pain centralization independent of peripheral nerve status.

### 4.4 Bilateral pain: evidence for supraspinal sensitization

Unilateral sciatic nerve injury in both ACI and CCI mice induced prominent contralateral mirror-image pain, a well-documented phenomenon across diverse preclinical pain models and multiple human chronic pain disorders (Arguis et al., 2008; Huang and Yu, 2010; Mathew et al., 2007; Shenker et al., 2008). In both experimental models, contralateral mechanical allodynia initially emerged at day 9, peaked stably at day 30, and persisted continuously until the 250-day endpoint. Despite fundamentally distinct peripheral pathological outcomes, ACI and CCI mice exhibited highly consistent bilateral pain progression patterns. This key observation makes it unlikely that sustained peripheral nociceptive input as the core driver of contralateral hypersensitivity. Combined with the delayed onset of contralateral pain relative to ipsilateral pain, these temporal and behavioral features are consistent with progressive cross-spinal signal transmission and widespread supraspinal network sensitization, supporting that supraspinal neural mechanisms are critically involved in the generation and long-term maintenance of bilateral neuropathic pain after transient nerve injury.

### 4.5 BOLD-fMRI reveals divergent central reorganization trajectories

Longitudinal BOLD-fMRI imaging dynamically revealed divergent central neural reorganization during the transition from acute to chronic pain in the two models. At the early acute stage (day 3), ACI mice presented significantly attenuated BOLD signals across cortical, subcortical and cerebellar regions compared with CCI mice, reflecting weaker central nociceptive activation induced by transient and reversible nerve compression. In contrast, persistent peripheral nerve damage in CCI mice was associated with robust activation of the cerebellar vermis, a key brain region mediating nociceptive transduction, sensory integration and pain-related emotional regulation (Manda et al., 2025). At day 12, CCI mice exhibited extensive unilateral cortical activation covering multiple sensory and functional cortices, consistent with peripheral nociception-driven cortical remodeling (Baliki et al., 2011; Quintero, 2013). Insular activation was detected in CCI mice. The insula serves as a core hub for affective and interoceptive pain processing and is tightly coupled to both sensory and motivational pain dimensions (Craig, 2009; Wiech et al., 2010). Conversely, ACI mice showed specific activation of hypothalamic nuclei, suggesting preferential engagement of stress-autonomic circuits rather than canonical nociceptive pathways, representing an early central adaptive response to the uncoupling of peripheral recovery and persistent pain.

At the chronic endpoint (day 250), the two models exhibited markedly different brain activation patterns. ACI mice displayed widespread bilateral activation throughout cortical, subcortical, midbrain, brainstem and major white matter tracts, while CCI mice showed restricted regional deactivation concentrated in the striatal complex, olfactory cortex and extended amygdala. The gradual expansion of activated brain regions in ACI mice reflects an evident pain centralization process, in which pain maintenance shifts from peripheral input-dependent activation to self-sustaining central network activity. The persistent activation of brainstem descending modulatory nuclei in late-stage ACI mice is consistent with a contribution of descending modulation (Basbaum and Fields, 1984; Mason, 2012; Porreca et al., 2002; Suzuki et al., 2004). Furthermore, pain-evoked responses in hippocampal formation subfields were greater in ACI than in CCI mice at day 250 (Supplementary Table 9; Figure 11I), which may provide a candidate neural substrate for their persistent anxiety-like behaviors (Kalisch et al., 2024; Madison et al., 2024; Turner and Onysk, 2022).

### 4.6 Affective comorbidities: temporal dissociation between pain and anxiety

ACI and CCI mice exhibited comparable pain hypersensitivity throughout the observation period, yet displayed markedly different long-term affective comorbidity outcomes. Both groups showed similar anxiety-like behaviors at day 12, which is consistent with the well-established clinical and preclinical comorbidity between neuropathic pain and emotional dysfunction (Dellarole et al., 2014; Yalcin et al., 2014). As shown by the OFT results, ACI mice maintained marked anxiety-like behavior at 7 months post-injury, whereas CCI mice had recovered to baseline levels. With pain load comparable between the two groups, this difference in affective comorbidity may not be attributable to pain itself, but rather to how the pain is driven: peripheral versus central drive.

In CCI mice, the persistent nerve injury provides a continuous and predictable nociceptive input that remains congruent with the pain percept, which may permit adaptive changes or habituation to develop within affective pain circuits. This accords with the striatal and extended-amygdala deactivation at day 250 (Supplementary Table 7; Figure 11G) and the recovery of open field behavior at day 215. Together with the early cortical and insular activation observed in CCI mice at day 12 (Supplementary Table 4; Figure 11D), this trajectory of early engagement followed by late disengagement resembles the adaptive stress resilience displayed by neural systems repeatedly exposed to challenge (Kalisch et al., 2024). By contrast, in ACI mice peripheral structure and function have markedly recovered while central pain persists. Ascending afferents can therefore no longer predict the pain state, giving rise to a cognitive-emotional conflict between “markedly recovered peripheral sensory feedback” and “ongoing central pain perception.” This sustained mismatch between afferent information and the central generative model of the body may amplify self-referential predictive beliefs and promote anxiety (Paulus and Stein, 2010; Van den Bergh et al., 2017). Two observations from the present study are consistent with this framework. First, the insular and hippocampal systems that encode pain salience and pain context (Craig, 2009; Turner and Onysk, 2022; Wiech et al., 2010) were precisely the regions in which the two models diverged at the chronic endpoint: at day 250, pain-evoked responses in the agranular insular cortex (bilaterally) and in hippocampal formation subfields (CA1, dentate gyrus, entorhinal and subicular areas) were greater in ACI than in CCI mice, with the insular difference being among the largest effects detected (Supplementary Table 9; Figure 11I). Second, this divergence reflects the loss of peripheral–central congruency rather than an absolute increase in insular recruitment: relative to Control mice, ACI mice did not show significant insular or hippocampal activation (Supplementary Table 8; Figure 11H), whereas CCI mice displayed pronounced deactivation of immediately adjacent piriform, striatal and extended-amygdala regions (Supplementary Table 7; Figure 11G). The ACI–CCI difference therefore most likely reflects maintained limbic engagement in ACI together with relative disengagement in CCI. Because our imaging sampled only three discrete time points with evoked stimuli, we cannot determine whether these responses are tonically or persistently elevated; the present data indicate greater task-evoked recruitment of these systems at the chronic stage, which is nevertheless compatible with a self-reinforcing mismatch between reafferent input and the central pain representation. When the central representation of pain shifts from nociceptive to emotional circuits during chronification, such a mismatch may produce affective consequences (Hashmi et al., 2013; Baliki and Apkarian, 2015). Superimposed on this sensory mismatch, early hypothalamic activation (Supplementary Table 5; Figure 11E) and chronic descending facilitation from brainstem nuclei (Porreca et al., 2002; Suzuki et al., 2004) may sustain hypothalamic–pituitary-adrenocortical axis hyperactivity within a bidirectionally coupled limbic-hypothalamic-brainstem loop in which “negative affect and pain mutually reinforce” (Ulrich-Lai and Herman, 2009; Wiech and Tracey, 2009), and the resulting glucocorticoid load may further remodel pain- and emotion-related circuits in a self-sustaining manner (Blackburn-Munro and Blackburn-Munro, 2001; Kuner and Flor, 2017), consistent with the greater hippocampal pain-evoked responses observed in ACI than in CCI mice at day 250 (Supplementary Table 9; Figure 11I). It thus appears that the two models differ not in pain load but in whether peripheral afferent drive remains congruent with the central pain representation: CCI mice carry persistent peripheral pathology yet retain this congruency, whereas in ACI mice the congruency has been lost. Therefore, the persistent affective comorbidity in centralized pain may reflect the loss of peripheral-central congruency rather than peripheral nerve injury status itself, and restoring this congruency may have therapeutic significance.

### 4.7 Clinical implications

Findings from the ACI model may help refine our understanding of pain chronification, revealing a narrower therapeutic time window for chronic pain prevention than previously recognized. Even short-term transient nerve compression can trigger central sensitization and form self-sustaining chronic pain. Clinically, early and active intervention for acute nerve compression before the establishment of central sensitization may be critical to block pain chronification. Once pain centralization is formed, single peripheral treatments such as surgical decompression and nerve blocks may be insufficient, requiring combined therapeutic strategies targeting central pain mechanisms. Moreover, the persistent anxiety observed in centralized pain highlights the necessity of integrating affective comorbidity management into routine clinical pain treatment. The divergent central reorganization patterns between ACI and CCI mice also support mechanism-based individualized pain therapy. Neuroimaging biomarkers that can effectively distinguish pain dominated by central mechanisms, peripheral mechanisms, or mixed pathogenesis, may provide objective evidence for precise subtyping and targeted clinical intervention of neuropathic pain.

### 4.8 Limitations

Our study has several inherent limitations. First, all experiments were conducted exclusively in male mice, excluding potential sex differences in ACI-induced pain behaviors and central sensitization mechanisms (Bartley and Fillingim, 2013). Future studies incorporating female animals are required to verify sex-dependent effects. Second, although multimodal functional data support central sensitization as the core pain maintenance mechanism of ACI, the specific molecular and cellular circuits remain uncharacterized. Further validation via immunohistochemistry, *in vivo* electrophysiology and genetic manipulation is needed. Third, manual mechanical stimulation during fMRI recording may introduce minor intensity variability, though standardized operating protocols and blinded analysis largely minimized the influence of experimental bias. Fourth, the 250-day observation period is one of the longest in neuropathic pain research, yet whether ACI-induced persistent pain can endure beyond this timeframe remains unclear. Fifth, a systematic comparison of compression durations was not performed in the present study and warrants future investigation. Additionally, SEPs were elicited with a fixed stimulation intensity (1 mA), and afferent volley recordings were not performed to calibrate the activated fiber population in each animal. Although this intensity was selected based on established protocols for sciatic nerve SEP recordings in rodents (Huang et al., 2018; Zaforas et al., 2024), minor inter-animal differences in nerve–electrode contact cannot be entirely excluded, which may contribute to variability in SEP amplitude. Future studies should incorporate afferent volley monitoring and normalize stimulus intensity to the afferent threshold to further standardize stimulation across animals.

## 5 Conclusion

In summary, results from the novel ACI model show that transient reversible peripheral nerve compression elicits persistent neuropathic pain despite obvious peripheral nerve structural and functional recovery. These findings indicate that sustained peripheral nerve lesions may not be required for chronic neuropathic pain to persist, adding a nuance to the prevailing view and lending support to the pain centralization hypothesis. Critically, ACI model provides a valuable platform for exploring the mechanisms underlying acute-to-chronic pain transition and facilitates the development of targeted therapies to prevent and reverse pain centralization in clinical compressive neuropathy.

## Data Availability

The original contributions presented in the study are included in the article/Supplementary material, further inquiries can be directed to the corresponding authors.

## References

[ref1] Arguis M. Perez J. Martinez G. Ubre M. Gomar C. (2008). Contralateral neuropathic pain following a surgical model of unilateral nerve injury in rats. Reg. Anesth. Pain Med. 33, 211–216. doi: 10.1136/rapm-00115550-200805000-00004, 18433671

[ref2] Attal N. Bouhassira D. Colvin L. (2023). Advances and challenges in neuropathic pain: a narrative review and future directions. Br. J. Anaesth. 131, 79–92. doi: 10.1016/j.bja.2023.04.021, 37210279

[ref31] Baliki M. N. Schnitzer T. J. Bauer W. R. Apkarian A. V. (2011). Brain morphological signatures for chronic pain. PLoS One 6:e26010. doi: 10.1371/journal.pone.0026010 PMC3192794 22022493

[ref3] Baliki M. N. Apkarian A. V. (2015). Nociception, pain, negative moods, and behavior selection. Neuron 87, 474–491. doi: 10.1016/j.neuron.2015.06.005, 26247858 PMC4529956

[ref4] Baron R. Binder A. Wasner G. (2010). Neuropathic pain: diagnosis, pathophysiological mechanisms, and treatment. Lancet Neurol. 9, 807–819. doi: 10.1016/s1474-4422(10)70143-5, 20650402

[ref5] Bartley E. J. Fillingim R. B. (2013). Sex differences in pain: a brief review of clinical and experimental findings. Br. J. Anaesth. 111, 52–58. doi: 10.1093/bja/aet127, 23794645 PMC3690315

[ref6] Basbaum A. I. Bautista D. M. Scherrer G. Julius D. (2009). Cellular and molecular mechanisms of pain. Cell 139, 267–284. doi: 10.1016/j.cell.2009.09.028, 19837031 PMC2852643

[ref7] Basbaum A. I. Fields H. L. (1984). Endogenous pain control systems: brainstem spinal pathways and endorphin circuitry. Annu. Rev. Neurosci. 7, 309–338. doi: 10.1146/annurev.ne.07.030184.001521, 6143527

[ref8] Bennett G. J. Xie Y. K. (1988). A peripheral mononeuropathy in rat that produces disorders of pain sensation like those seen in man. Pain 33, 87–107. doi: 10.1016/0304-3959(88)90209-6, 2837713

[ref9] Blackburn-Munro G. Blackburn-Munro R. E. (2001). Chronic pain, chronic stress and depression: coincidence or consequence? J. Neuroendocrinol. 13, 1009–1023. doi: 10.1046/j.0007-1331.2001.00727.x, 11722697

[ref10] Casaril A. M. Gaffney C. M. Shepherd A. J. (2024). “Animal models of neuropathic pain,” in Neuropathic Pain, eds. J. Rosner and P. Karlsson (London: Academic Press), 339–401. doi: 10.1016/bs.irn.2024.10.004 39580217

[ref11] Chi H.-H. Lee J.-C. Chen C.-C. Chen S.-K. Yen C.-T. (2020). An index combining lost and remaining nerve fibers correlates with pain hypersensitivity in mice. Cells 9:2414. doi: 10.3390/cells9112414, 33158176 PMC7694241

[ref12] Cohen S. P. Vase L. Hooten W. M. (2021). Chronic pain: an update on burden, best practices, and new advances. Lancet 397, 2082–2097. doi: 10.1016/S0140-6736(21)00393-7, 34062143

[ref13] Craig A. D. (2009). How do you feel — now? The anterior insula and human awareness. Nat. Rev. Neurosci. 10, 59–70. doi: 10.1038/nrn2555, 19096369

[ref14] Decosterd I. Woolf C. J. (2000). Spared nerve injury: an animal model of persistent peripheral neuropathic pain. Pain 87, 149–158. doi: 10.1016/S0304-3959(00)00276-1, 10924808

[ref15] Dellarole A. Morton P. Brambilla R. Walters W. Summers S. Bernardes D. . (2014). Neuropathic pain-induced depressive-like behavior and hippocampal neurogenesis and plasticity are dependent on Tnfr1 signaling. Brain Behav. Immun. 41, 65–81. doi: 10.1016/j.bbi.2014.04.003, 24938671 PMC4167189

[ref16] Feng X. Niu L. Long M. Luo K. Huang X. Chen M. . (2021). Transcranial ultrasound stimulation of the anterior cingulate cortex reduces neuropathic pain in mice. Evid. Based Complement. Altern. Med. 2021, 1–14. doi: 10.1155/2021/6510383 PMC8741380 35003307

[ref17] Gopalsamy B. Sambasevam Y. Zulazmi N. A. Chia J. S. M. Omar Farouk A. A. Sulaiman M. R. . (2019). Experimental characterization of the chronic constriction injury-induced neuropathic pain model in mice. Neurochem. Res. 44, 2123–2138. doi: 10.1007/s11064-019-02850-0, 31376053

[ref18] Hargreaves K. Dubner R. Brown F. Flores C. Joris J. (1988). A new and sensitive method for measuring thermal nociception in cutaneous hyperalgesia. Pain 32, 77–88. doi: 10.1016/0304-3959(88)90026-7, 3340425

[ref19] Hashmi J. A. Baliki M. N. Huang L. Baria A. T. Torbey S. Hermann K. M. . (2013). Shape shifting pain: chronification of back pain shifts brain representation from nociceptive to emotional circuits. Brain 136, 2751–2768. doi: 10.1093/brain/awt211, 23983029 PMC3754458

[ref20] Ho Kim S. Mo Chung J. (1992). An experimental model for peripheral neuropathy produced by segmental spinal nerve ligation in the rat. Pain 50, 355–363. doi: 10.1016/0304-3959(92)90041-9, 1333581

[ref21] Huang S. Fisher J. A. N. Ye M. Kim Y.-S. Ma R. Nabili M. . (2018). Epidermal electrode Technology for Detecting Ultrasonic Perturbation of sensory brain activity. IEEE Trans. Biomed. Eng. 65, 1272–1280. doi: 10.1109/TBME.2017.2713647, 28858781 PMC9472568

[ref22] Huang D. Yu B. (2010). The mirror-image pain: an unclered phenomenon and its possible mechanism. Neurosci. Biobehav. Rev. 34, 528–532. doi: 10.1016/j.neubiorev.2009.10.011, 19883682

[ref23] Inserra M. M. Bloch D. A. Terris D. J. (1998). Functional indices for sciatic, peroneal, and posterior tibial nerve lesions in the mouse. Microsurgery 18, 119–124. doi: 10.1002/(SICI)1098-2752(1998)18:2<119::AID-MICR10>3.0.CO;2-0, 9674927

[ref24] Johnson G. A. Tian Y. Ashbrook D. G. Cofer G. P. Cook J. J. Gee J. C. . (2023). Merged magnetic resonance and light sheet microscopy of the whole mouse brain. Proc. Natl. Acad. Sci. USA 120:e2218617120. doi: 10.1073/pnas.2218617120, 37068254 PMC10151475

[ref25] Kalisch R. Russo S. J. Müller M. B. (2024). Neurobiology and systems biology of stress resilience. Physiol. Rev. 104, 1205–1263. doi: 10.1152/physrev.00042.2023, 38483288 PMC11381009

[ref26] Kuffler D. Reyes O. Sosa I. Micheo W. Santiago-Figueroa J. Foy C. (2025). Clinically reducing/eliminating chronic neuropathic pain by bridging peripheral nerve gaps with an autograft within a Prp-filled collagen tube. J. Pain Res. 18, 3207–3216. doi: 10.2147/JPR.S523451, 40599253 PMC12212433

[ref27] Kuner R. Flor H. (2017). Structural plasticity and reorganisation in chronic pain. Nat. Rev. Neurosci. 18, 113–113. doi: 10.1038/nrn.2017.5, 28704354

[ref28] Latremoliere A. Woolf C. J. (2009). Central sensitization: a generator of pain hypersensitivity by central neural plasticity. J. Pain 10, 895–926. doi: 10.1016/j.jpain.2009.06.012, 19712899 PMC2750819

[ref29] Liao Y.-H. Chen M.-X. Chen S.-C. Luo K.-X. Wang B. Liu Y. . (2021). Effects of noninvasive low-intensity focus ultrasound neuromodulation on spinal cord neurocircuits in vivo. Evid. Based Complement. Alternat. Med. 202, 1–16. 10.1155/2021/8534466 PMC8643243 34873411

[ref30] Luo Y. Luo K. Zhang X. Feng X. Lei L. Cheng S. . (2025). Low-intensity focused ultrasound stimulation on the hippocampus improved Csds-induced neuroinflammation through the Notch1 signaling pathway. J. Affect. Disord. 390:119793. doi: 10.1016/j.jad.2025.119793, 40602537

[ref32] Madison F. N. Bingman V. P. Smulders T. V. Lattin C. R. (2024). A bird's eye view of the hippocampus beyond space: behavioral, neuroanatomical, and neuroendocrine perspectives. Horm. Behav. 157:105451. doi: 10.1016/j.yhbeh.2023.105451, 37977022

[ref33] Manda O. Hadjivassiliou M. Varrassi G. Zavridis P. Zis P. (2025). Exploring the role of the cerebellum in pain perception: a narrative review. Pain Ther. 14, 803–816. doi: 10.1007/s40122-025-00724-8, 40097893 PMC12085741

[ref34] Mason P. (2012). Medullary circuits for nociceptive modulation. Curr. Opin. Neurobiol. 22, 640–645. doi: 10.1016/j.conb.2012.03.008, 22483535 PMC4548289

[ref35] Mathew N. T. Kailasam J. Meadors L. (2007). Predictors of response to botulinum toxin type a (Bonta) in chronic daily headache. Headache 48, 194–200. doi: 10.1111/j.1526-4610.2007.00914.x, 17868356

[ref36] Middleton S. D. Anakwe R. E. (2014). Carpal tunnel syndrome. BMJ 349:g6437. doi: 10.1136/bmj.g6437 25378457

[ref37] Paulus M. P. Stein M. B. (2010). Interoception in anxiety and depression. Brain Struct. Funct. 214, 451–463. doi: 10.1007/s00429-010-0258-9, 20490545 PMC2886901

[ref38] Porreca F. Ossipov M. H. Gebhart G. F. (2002). Chronic pain and medullary descending facilitation. Trends Neurosci. 25, 319–325. doi: 10.1016/S0166-2236(02)02157-4, 12086751

[ref39] Quintero G. (2013). Advances in cortical modulation of pain. J. Pain Res. 6, 713–725. doi: 10.2147/JPR.S45958, 24092997 PMC3788691

[ref40] Rodríguez-Merchán E. C. Moracia-Ochagavía I. (2021). Tarsal tunnel syndrome: current rationale, indications and results. Efort Open Rev. 6, 1140–1147. doi: 10.1302/2058-5241.6.210031, 35839088 PMC8693231

[ref41] Shenker N. G. Haigh R. C. Mapp P. I. Harris N. Blake D. R. (2008). Contralateral hyperalgesia and allodynia following intradermal capsaicin injection in man. Rheumatology 47, 1417–1421. doi: 10.1093/rheumatology/ken251, 18632788 PMC2518944

[ref42] Soliman N. Moisset X. Ferraro M. C. De Andrade D. C. Baron R. Belton J. . (2025). Pharmacotherapy and non-invasive neuromodulation for neuropathic pain: a systematic review and meta-analysis. Lancet Neurol. 24, 413–428. doi: 10.1016/S1474-4422(25)00068-7, 40252663

[ref43] Suzuki R. Rygh L. J. Dickenson A. H. (2004). Bad news from the brain: descending 5-Ht pathways that control spinal pain processing. Trends Pharmacol. Sci. 25, 613–617. doi: 10.1016/j.tips.2004.10.002, 15530638

[ref44] Toci G. R. Lambrechts M. J. Karamian B. A. Canseco J. A. Hilibrand A. S. Kepler C. K. . (2023). Patients with radiculopathy have worse baseline disability and greater improvements following anterior cervical discectomy and fusion compared to patients with myelopathy. Spine J. 23, 238–246. doi: 10.1016/j.spinee.2022.10.005, 36257530

[ref45] Turner G. Onysk J. (2022). The Hippocampus may support context retrieval in one-shot learning about pain. J. Neurosci. 42, 1883–1885. doi: 10.1523/JNEUROSCI.2250-21.2022, 35264429 PMC8916767

[ref46] Ulrich-Lai Y. M. Herman J. P. (2009). Neural regulation of endocrine and autonomic stress responses. Nat. Rev. Neurosci. 10, 397–409. doi: 10.1038/nrn2647, 19469025 PMC4240627

[ref47] Van Den Bergh O. Witthöft M. Petersen S. Brown R. J. (2017). Symptoms and the body: taking the inferential leap. Neurosci. Biobehav. Rev. 74, 185–203. doi: 10.1016/j.neubiorev.2017.01.015, 28108416

[ref48] Van Hecke O. Austin S. K. Khan R. A. Smith B. H. Torrance N. (2014). Neuropathic pain in the general population: a systematic review of epidemiological studies. Pain 155, 654–662. doi: 10.1016/j.pain.2013.11.013, 24291734

[ref49] Wiech K. Lin C.-S. Brodersen K. H. Bingel U. Ploner M. Tracey I. (2010). Anterior insula integrates information about salience into perceptual decisions about pain. J. Neurosci. 30, 16324–16331. doi: 10.1523/JNEUROSCI.2087-10.2010, 21123578 PMC6634837

[ref50] Wiech K. Tracey I. (2009). The influence of negative emotions on pain: behavioral effects and neural mechanisms. NeuroImage 47, 987–994. doi: 10.1016/j.neuroimage.2009.05.059, 19481610

[ref51] Woolf C. J. (2011). Central sensitization: implications for the diagnosis and treatment of pain. Pain 152, S2–S15. doi: 10.1016/j.pain.2010.09.030, 20961685 PMC3268359

[ref52] Yalcin I. Barthas F. Barrot M. (2014). Emotional consequences of neuropathic pain: insight from preclinical studies. Neurosci. Biobehav. Rev. 47, 154–164. doi: 10.1016/j.neubiorev.2014.08.002, 25148733

[ref53] Zaforas M. Miguel-Quesada C. Fernández-López E. Alonso-Calviño E. Barranco-Maresca V. Misol-Ortiz A. . (2024). Protocol for stimulating specific rodent limb receptive fields while recording in vivo somatosensory-evoked activity. Star Prot. 5:102972. doi: 10.1016/j.xpro.2024.102972, 38502685 PMC10960094

[ref9001] Zimmermann M. (1983). Ethical guidelines for investigations of experimental pain in conscious animals. Pain 16, 109–110. doi: 10.1016/0304-3959(83)90201-4, 6877845

