# Supplementary material for: Transient peripheral compression induces persistent central pain: the acute constriction injury model
Source: Front Syst Neurosci. 2026 Oct 26;20:1962461. doi: 10.3389/fnsys.2026.1962461 (PMC13745477; doi:10.3389/fnsys.2026.1962461)
Supplement: Supplementary file 1 [file Data_Sheet_1.PDF]

Supplementary Table 1. BOLD responses to right hindpaw mechanical pain stimulation at 3 d post-operation: CCI vs Control

Brain region	Number of significant voxels	Peak T value	Allen coordinate (mm)
Vermal regions, left	286	4.97	1.09, 11.38, -16.54

Note: Data were analyzed using two-sample t-tests with Gaussian random field (GRF) correction (voxel $P < 0.005$, cluster $P < 0.05$). n=7 per group.

Supplementary Table 2. BOLD responses to right hindpaw mechanical pain stimulation at 3 d post-operation: ACI vs Control

Brain region	Number of significant voxels	Peak T value	Allen coordinate (mm)
Temporal association areas, left	117	-3.75	3.89, 11.33, -13.39
Primary visual area, left	1230	-4.59	3.24, 11.63, -14.19

Note: Data were analyzed using two-sample t-tests with Gaussian random field (GRF) correction (voxel $P < 0.005$, cluster $P < 0.05$). n=7 per group.

Supplementary Table 3. BOLD responses to right hindpaw mechanical pain stimulation at 3 d post-operation: ACI vs CCI

Brain region	Number of significant voxels	Peak T value	Allen coordinate (mm)
Dorsal hippocampal commissure, left	388	-2.36	2.94, 11.18, -13.79
Prelimbic area, left	726	-2.78	0.79, 11.73, -6.94
Anterior cingulate area, left	3674	-3.09	0.79, 11.78, -6.99
Primary motor area, left	527	-2.75	1.04, 11.98, -10.29
Secondary motor area, left	2740	-3.20	0.89, 12.03, -7.04
Primary somatosensory area, left	142	-2.43	1.04, 12.18, -10.64
Retrosplenial area, left	1741	-2.60	0.64, 12.03, -10.49
Visual areas, left	3454	-3.50	3.74, 11.23, -14.24
Primary visual area, left	375	-2.47	2.39, 11.68, -13.84
Entorhinal area, left	2424	-3.10	3.74, 10.98, -14.34
Subicular region, left	2701	-2.79	3.69, 11.08, -14.64
Hippocampus, left	240	-3.04	1.24, 11.63, -10.69
Field CA1, left	1714	-3.04	0.89, 11.73, -10.69

Field CA3, left	2241	-3.11	1.09, 11.53, -10.64
Dentate gyrus, left	648	-2.90	0.84, 11.48, -10.64
Lateral septal nucleus, left	178	-2.12	0.49, 11.33, -9.79
Anterodorsal nucleus, left	103	-2.08	0.99, 10.83, -10.09
Lateral dorsal nucleus of the thalamus, left	169	-2.12	1.04, 10.88, -10.14
Superior colliculus sensory related, left	451	-2.13	1.64, 11.68, -13.74
Superior colliculus motor related, left	1781	-2.28	1.49, 11.68, -13.74
Inferior colliculus, left	608	-2.24	1.99, 11.48, -14.29
Pretectal region, left	369	-2.06	0.59, 11.48, -12.44
Interposed nucleus, left	106	-2.12	1.89, 9.93, -15.44
Cingulum bundle, left	778	-2.79	0.64, 12.03, -10.69
Anterior cingulate area, right	2597	-2.79	-0.41, 11.98, -7.49
Agranular insular area, right	1147	-3.15	-3.91, 9.68, -8.14
Gustatory areas, right	2002	-4.23	-4.31, 9.58, -8.69
Piriform area, right	1644	-2.60	-4.31, 8.63, -10.14
Visceral area, right	545	-3.38	-4.41, 9.78, -9.04
Primary motor area, right	5297	-3.28	-1.91, 11.53, -7.89
Primary somatosensory area, right	6323	-2.92	-2.06, 11.68, -9.09
Retrosplenial area, right	347	-2.12	-0.11, 11.83, -10.24
Subicular region, right	213	-2.08	-1.71, 11.63, -13.64
Striatum dorsal region, right	407	-2.25	-2.16, 11.33, -9.44
Superior colliculus sensory related, right	337	-2.32	-1.61, 11.63, -13.74
Inferior colliculus, right	652	-2.18	-1.46, 10.88, -14.09
Inferior colliculus central nucleus, right	267	-2.27	-1.06, 10.78, -14.59
Cuneiform nucleus, right	134	-2.23	-1.06, 10.68, -14.59
Parabrachial nucleus, right	140	-2.26	-1.11, 9.63, -15.14
Vermal regions, right	1834	-2.24	-1.56, 10.13, -15.09
Interposed nucleus, right	106	-2.08	-1.81, 10.03, -15.39
Vestibular nuclei, right	655	-2.68	-1.51, 9.43, -15.59
Corpus callosum, right	1138	-2.40	-1.96, 11.53, -9.44

Note: Data were analyzed using two-sample t-tests with Gaussian random field (GRF) correction (voxel $P < 0.005$, cluster $P < 0.05$). n=7 per group.

Supplementary Table 4. BOLD responses to right hindpaw mechanical pain stimulation at 12 d post-operation: CCI vs Control

Brain region	Number of significant voxels	Peak T value	Allen coordinate (mm)
Olfactory bulb, left	2500	-5.01	0.59, 11.18, -4.04

Retrosplenial area, left	487	3.91	1.99, 11.73, -14.29
Inferior colliculus central nucleus, left	1883	4.40	1.19, 11.53, -15.14
Vermal regions, left	13903	5.38	0.39, 11.53, -15.99
Agranular insular area, right	2039	5.63	-3.56, 9.83, -7.99
Gustatory areas, right	3618	7.67	-3.46, 10.33, -7.44
Primary motor area, right	5109	7.37	-3.36, 10.33, -7.44
Primary somatosensory area, right	13945	8.34	-3.21, 11.28, -8.14
Primary somatosensory area barrel field, right	1286	4.67	-3.61, 11.98, -10.19
Supplemental somatosensory area, right	3668	5.21	-3.81, 10.28, -8.19
Auditory areas, right	507	3.92	-4.66, 11.43, -11.14
Retrosplenial area, right	285	3.89	-2.36, 11.88, -14.39
Primary visual area, right	5273	8.92	-2.91, 12.08, -13.84
Vermal regions, right	6144	4.87	-1.21, 11.53, -17.14

Note: Data were analyzed using two-sample t-tests with Gaussian random field (GRF) correction (voxel *P* < 0.005, cluster *P* < 0.05). n=7 per group.

Supplementary Table 5. BOLD responses to right hindpaw mechanical pain stimulation at 12 d post-operation: ACI vs Control

Brain region	Number of significant voxels	Peak T value	Allen coordinate (mm)
Hypothalamus, left	996	4.44	0.84, 7.68, -10.84
Ventromedial hypothalamic nucleus, left	640	4.28	0.84, 7.53, -10.84
Tuberal nucleus, left	577	3.98	1.29, 7.68, -10.79

Note: Data were analyzed using two-sample t-tests with Gaussian random field (GRF) correction (voxel *P* < 0.005, cluster *P* < 0.05). n=7 per group.

Supplementary Table 6. BOLD responses to right hindpaw mechanical pain stimulation at 12 d post-operation: ACI vs CCI

Brain region	Number of significant voxels	Peak T value	Allen coordinate (mm)
Agranular insular area, right	273	-3.64	-3.76, 9.78, -8.04
Gustatory areas, right	2344	-4.48	-3.61, 10.08, -8.44
Primary somatosensory area, right	380	-3.97	-3.36, 10.18, -8.44
Supplemental somatosensory area, right	3052	-4.46	-3.56, 10.18, -9.04
Auditory areas, right	1745	-4.95	-4.91, 10.93, -12.74

Temporal association areas, right	386	-4.50	-4.76, 10.98, -13.24
Striatum dorsal region, right	429	-4.08	-3.36, 10.23, -9.04

Note: Data were analyzed using two-sample t-tests with Gaussian random field (GRF) correction (voxel $P < 0.005$, cluster $P < 0.05$). n=7 per group.

Supplementary Table 7. BOLD responses to right hindpaw mechanical pain stimulation at 250 d post-operation: CCI vs Control

Brain region	Number of significant voxels	Peak T value	Allen coordinate (mm)
Piriform area, left	3874	-5.03	3.39, 8.08, -8.54
Endopiriform nucleus, left	102	-3.78	2.94, 8.63, -8.29
Striatum dorsal region, left	2736	-4.29	2.29, 9.03, -9.19
Fundus of striatum, left	154	-4.23	2.59, 8.33, -8.74
Bed nuclei of the stria terminalis, left	197	-3.63	1.09, 9.53, -9.14
Endopiriform nucleus, right	386	-3.65	-2.31, 8.83, -7.79
Striatum dorsal region, right	749	-3.81	-1.26, 9.28, -8.34
Nucleus accumbens, right	1204	-3.87	-1.01, 9.28, -7.94
Olfactory tubercle, right	229	-3.93	-2.11, 7.98, -7.39
anterior commissure, right	161	-3.69	-1.21, 9.03, -7.94

Note: Data were analyzed using two-sample t-tests with Gaussian random field (GRF) correction (voxel $P < 0.005$, cluster $P < 0.05$). n=7 per group.

Supplementary Table 8. BOLD responses to right hindpaw mechanical pain stimulation at 250 d post-operation: ACI vs Control

Brain region	Number of significant voxels	Peak T value	Allen coordinate (mm)
Pons, left	134	4.87	0.09, 7.23, -14.14
Facial motor nucleus, left	2513	5.32	1.39, 7.13, -15.34
Pontine reticular formation, left	1727	5.03	0.09, 7.23, -14.19
Inferior olivary complex, left	287	4.68	0.14, 6.93, -15.69
Medullary reticular zone, left	10058	6.05	0.49, 6.93, -14.94
Intermediate reticular nucleus, left	4450	5.66	0.99, 7.53, -14.89
Medulla raphe behavioral state related, left	579	6.31	-0.01, 7.18, -14.29
corticospinal tract, left	695	6.1	0.04, 7.18, -14.34
Hypothalamus, right	261	5.43	-1.01, 7.78, -11.74

Mammillary body, right	623	9.48	-1.01, 7.98, -12.04
Lateral hypothalamic area, right	218	5.978	-1.21, 8.08, -11.74
Substantia nigra reticular part, right	175	6.12	-1.16, 8.28, -12.19
Pons, right	828	5.16	-0.21, 8.78, -14.89
Principal sensory nucleus of the trigeminal, right	199	4.51	-2.01, 7.88, -14.64
Facial motor nucleus, right	2014	5.85	-1.31, 7.23, -15.09
Pontine reticular formation, right	4249	7.50	-0.36, 7.18, -14.14
Spinal nucleus of the trigeminal region, right	826	4.33	-1.71, 8.33, -15.24
Ventral cochlear nucleus, right	333	5.29	-2.46, 7.98, -15.09
Vestibular nuclei, right	134	4.57	-0.56, 9.13, -15.29
Inferior olivary complex, right	126	4.60	-0.16, 6.93, -15.69
Medullary reticular zone, right	10883	6.06	-0.21, 7.18, -14.44
Intermediate reticular nucleus, right	4317	5.36	-1.21, 7.48, -15.24
Medulla raphe behavioral state related, right	274	6.73	-0.06, 7.18, -14.29
corticospinal tract, right	1523	9.70	-1.21, 8.23, -12.39

Note: Data were analyzed using two-sample t-tests with Gaussian random field (GRF) correction (voxel $P < 0.005$, cluster $P < 0.05$). n=7 per group.

Supplementary Table 9. BOLD responses to right hindpaw mechanical pain stimulation at 250 d post-operation: ACI vs CCI

Brain region	Number of significant voxels	Peak T value	Allen coordinate (mm)
Olfactory bulb, left	207	4.73	1.84, 9.58, -6.19
Anterior olfactory nucleus, left	906	4.27	2.04, 9.68, -6.59
Orbital area, left	927	4.267	2.09, 10.08, -6.89
Agranular insular area, left	8841	8.34	2.54, 9.88, -7.24
Gustatory areas, left	223	4.20	3.44, 10.08, -7.29
Piriform area, left	2804	5.50	2.29, 9.53, -6.79
Primary motor area, left	2353	5.60	2.39, 11.33, -8.59
Primary somatosensory area, left	2175	8.02	2.74, 11.08, -8.59
Subicular region, left	648	4.94	2.59, 10.53, -14.14
Claustrum, left	493	5.30	2.64, 9.83, -7.59
Endopiriform nucleus, left	181	4.06	3.34, 8.58, -8.89
Striatum, left	496	4.10	1.29, 9.03, -8.79
Striatum dorsal region, left	5651	4.81	2.44, 9.93, -7.79
Fundus of striatum, left	442	3.85	2.14, 8.33, -9.29
Globus pallidus external segment, left	140	3.65	1.44, 9.13, -9.39

Substantia innominata, left	2153	4.26	1.59, 8.38, -9.79
Lateral septal nucleus, left	639	4.00	0.04, 10.83, -9.09
Bed nuclei of the stria terminalis, left	3613	5.03	1.04, 9.23, -9.59
Thalamus, left	627	4.99	0.99, 9.28, -9.69
Anteromedial nucleus, left	724	5.20	0.84, 9.38, -9.99
Ventral anterior-lateral complex of the thalamus, left	166	4.20	0.89, 9.58, -10.29
Ventral medial nucleus of the thalamus, left	908	5.21	0.89, 9.38, -9.99
Reticular nucleus of the thalamus, left	962	5.32	0.99, 9.28, -9.84
Hypothalamus, left	1968	5.53	1.09, 8.88, -9.84
Anterior hypothalamic nucleus, left	246	4.11	0.89, 8.13, -9.84
Lateral preoptic area, left	732	4.75	1.19, 8.58, -9.44
Lateral hypothalamic area, left	3664	6.27	1.19, 8.38, -9.74
Zona incerta, left	938	5.45	1.04, 8.88, -9.89
Inferior colliculus, left	307	4.08	2.19, 10.48, -13.99
Pedunculopontine nucleus, left	139	3.80	1.74, 9.73, -14.19
Cuneiform nucleus, left	283	4.25	1.79, 10.08, -14.09
Nucleus of the lateral lemniscus, left	105	5.77	2.04, 9.98, -14.19
Vermal regions, left	5983	5.34	1.79, 11.03, -15.74
corpus callosum, left	1069	6.47	2.89, 10.83, -8.59
anterior commissure, left	704	4.14	1.94, 8.83, -9.14
columns of the fornix, left	231	4.31	0.89, 8.38, -9.94
Orbital area, right	144	4.30	-1.96, 9.98, -6.99
Anterior cingulate area, right	1042	4.77	-0.46, 11.63, -9.39
Agranular insular area, right	6460	5.52	-2.86, 10.08, -7.14
Gustatory areas, right	584	6.45	-3.51, 10.38, -7.34
Piriform area, right	7062	6.99	-2.56, 8.13, -7.74
Primary motor area, right	2055	7.27	-3.46, 10.38, -7.24
Primary somatosensory area, right	1356	7.05	-3.61, 10.58, -7.39
Primary somatosensory area barrel field, right	136	3.95	-3.41, 11.88, -12.04
Supplemental somatosensory area, right	138	4.52	-3.61, 11.88, -12.04
Auditory areas, right	1390	4.79	-3.61, 11.68, -12.24
Temporal association areas, right	113	3.86	-4.41, 10.98, -13.04
Retrosplenial area, right	396	3.90	-1.76, 12.08, -13.64
Primary visual area, right	1431	4.39	-2.26, 12.08, -13.19
Entorhinal area, right	1524	4.58	-3.21, 10.93, -14.34
Subicular region, right	1074	3.99	-3.01, 10.58, -13.64
Field CA1, right	618	4.29	-3.11, 11.23, -12.19
Dentate gyrus, right	381	3.91	-2.76, 10.58, -13.39
Claustrum, right	512	4.35	-2.61, 9.58, -7.74
Endopiriform nucleus, right	2072	4.91	-2.61, 8.93, -7.79
Striatum, right	360	4.11	-0.31, 10.78, -8.44

Striatum dorsal region, right	2884	5.25	-1.51, 11.43, -9.49
Nucleus accumbens, right	402	4.44	-2.21, 8.43, -8.09
Olfactory tubercle, right	1165	7.07	-2.51, 8.08, -7.74
Substantia innominata, right	181	4.26	-1.96, 8.13, -7.74
Lateral septal nucleus, right	1672	4.54	-0.16, 10.63, -9.09
Vermal regions, right	2397	4.37	-0.31, 11.48, -15.79
Floccular nodular region, right	169	4.37	-0.46, 9.78, -17.99
corpus callosum, right	2328	5.14	-0.41, 11.43, -9.59
cingulum bundle, right	201	5.11	-0.81, 11.43, -8.94
dorsal hippocampal commissure, right	289	4.20	-2.06, 11.88, -13.39

Note: Data were analyzed using two-sample t-tests with Gaussian random field (GRF) correction (voxel $P < 0.005$, cluster $P < 0.05$). n=7 per group.
